# Concise Large-Scale Synthesis of Tomatidine, A Potent Antibiotic Natural Product

**DOI:** 10.3390/molecules26196008

**Published:** 2021-10-03

**Authors:** Chad Normandin, Pierre-Luc Boudreault

**Affiliations:** Institut de Pharmacologie, Université of Sherbrooke, Sherbrooke, QC J1H 5N4, Canada; cnorm102@uottawa.ca

**Keywords:** tomatidine, antibiotics, alkaloids, steroids, *Staphylococcus aureus*, small-colony variants, large-scale

## Abstract

Tomatidine has recently generated a lot of interest amongst the pharmacology, medicine, and biology fields of study, especially for its newfound activity as an antibiotic agent capable of targeting multiple strains of bacteria. In the light of its low natural abundance and high cost, an efficient and scalable multi-gram synthesis of tomatidine has been developed. This synthesis uses a Suzuki–Miyaura-type coupling reaction as a key step to graft an enantiopure F-ring side chain to the steroidal scaffold of the natural product, which was accessible from low-cost and commercially available diosgenin. A Lewis acid-mediated spiroketal opening followed by an azide substitution and reduction sequence is employed to generate the spiroaminoketal motif of the natural product. Overall, this synthesis produced 5.2 g in a single pass in 15 total steps and 15.2% yield using a methodology that is atom economical, scalable, and requires no flash chromatography purifications.

## 1. Introduction

The genus *Solanum* consists of over 2000 species of plants widely distributed over the world, from which over 600 natural products have been isolated [1]. Ten percent of these products are alkaloid steroids, which are classified into the *Veratrum* and *Solanum* alkaloid families [2]. Tomatidine (TO, **1**), which belongs to the solanidine subfamily of *Solanum*, has recently gathered great interest from the scientific community for its biological and medicinal applications stemming from its anabolic [3], antioxidant [4], neuroprotective [5], antiviral [6,7], anticancer [8], and most importantly, antibiotic [9,10,11,12,13] properties. Our research group originally discovered the antibiotic potential of **1** against *Staphylococcus aureus* and its virulent small-colony variants (SCV), which are responsible for resistance and persistence of the pathogen [9,10]. Structure–activity relationship (SAR) studies based on modifications of the natural product showed that it was possible to bring the minimum inhibitory concentration (MIC) of *S. aureus* from >128 μg/mL to 2 μg/mL using the most active derivative FC04-100 [11,12]. Furthermore, the MIC could be brought down from 64 μg/mL to 8 μg/mL for the SCVs (8-fold potency increase) when TO was used synergistically with the aminoglycoside antibiotic gentamycin [12].

Accessibility is a challenge commonly encountered in the study of biologically active natural products [14]. Recently, our group has shown that the potent alkaloid tomatidine could be synthesized on gram-scale [15], with only one other methodology producing a close derivative (tomatidenol, ^Δ5-6^-**1**) [16] in small quantities (50 mg) through non-strategic redox [17]. With SAR studies still on-going in our laboratory and considering the common profound scientific interest, high costs (> USD 1500/g) and scarce natural availability of this steroid, we tackled the ambitious challenge of developing a new synthesis capable of decagram scale quantities of **1**.

**Scheme 1:**
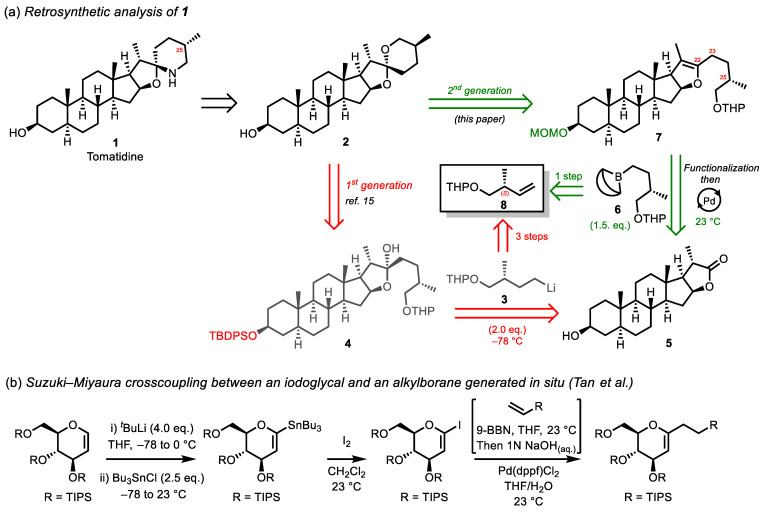
(**a**) Retrosynthetic analysis of Tomatidine and (**b**) proposed coupling methodology.

Despite providing over 2 g of tomatidine, we identified multiple shortcomings in our previous synthetic methodology that would render a scale-up largely inefficient. Our previous methodology (Scheme 1a, red) [15] relied on a chiral auxiliary to set the stereocenter of iodo-**3**, followed by its introduction to **5** as a temperature-sensitive organolithium reagent to obtain adduct **4**, which then underwent acid-mediated spiroketalization to obtain **2**. To develop a scalable and atom-economical synthesis, inefficient steps of the first-generation synthesis, such as the use of the heavy TBDPS protecting group, requirement of a chiral auxiliary, and the arduous temperature control of the organolithium **3** generation on higher scales, had to be addressed. To further optimize the overall practicality of this methodology, we also set the ambitious objective of avoiding the use of flash chromatography purifications for the whole sequence, aiming to develop clean transformations and to obtain pure intermediates from recrystallizations, distillations, and silica pads only.

We recognized early in our new retrosynthetic analysis (Scheme 1a, green) that the C_22_–C_23_ bond was an efficient disconnection strategy for introducing a side chain synthon containing the required (*S*)-C_25_ of the natural product. We opted to use the Suzuki–Miyaura reaction to combine the steroid scaffold **5** to the required asymmetric synthon **8** (as organoborane **6**). This reaction is known to be a generally efficient and cost-effective method to give access to pharmaceutical intermediates and natural products even on industrial scales [18]. The union of an iodo enol ether (as iodo dihydropyran) to an alkylborane generated in situ from a parent olefin through Pd catalysis has been previously demonstrated by Tan et al. [19] to afford the desired adducts in high yields (Scheme 1,b). We planned to use and optimize this methodology by circumventing the use of the stannyl intermediate in the preparation of the required iodo enol ether. Succeeding at this critical step would, following cyclisation, lead us to spirostanol **2**, which would lead us to tomatidine **1** through our previously developed sequence of five reactions [15].

Preparing the required enol ether following this strategy would start from functionalization of lactone **5**, followed by coupling to alkylborane **6** to obtain adduct **7**, which, after an acid-catalyzed spiroketalization, would deliver **2**. This approach requires less of the valuable asymmetric synthon **8** (1.5 eq., vs. 2.0 eq. required in the first-generation synthesis) and avoids the temperature dependency of its union to the steroid backbone. Scaffold **5** could be obtained in two steps on large scale from diosgenin [20], an inexpensive (USD 0.30/g) sapogenin, obtained mainly from the acidic hydrolysis of wild yam (*Dioscorea*) on industrial scale [21].

## 2. Results and Discussion

**Scheme 2:**
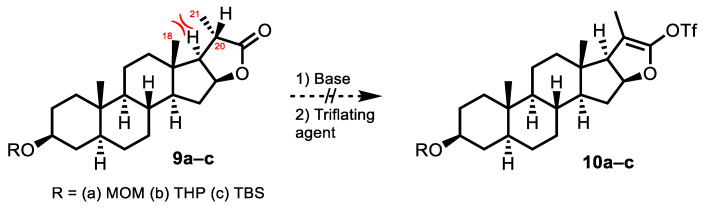
Failed attempts at obtaining triflates **10a–c**.

Our efforts started with the preparation of the first anchor for the Pd-mediated Suzuki–Miyaura reaction, the steroid core. We initially probed the reactivity of lactone **5** as its C_3_-protected congeners **9a–c** (Scheme 2) in the hope of obtaining enol triflates **10a–c**, which would give access to the required coupling partner following our retrosynthetic strategy. Conversion of ketones and lactones to their corresponding enol triflates followed by cross-coupling is a strategic way to access diverse complex natural products in a convergent manner [22]. Despite extensive efforts, lactones **9a–c** resisted triflation under common conditions (LiHMDS/KHMDS/LDA then Ph_2_NTf/Tf_2_O/Comin’s reagent) [22,23], most likely because of the strong steric hindrance imparted by the C_18_ and C_21_ methyl groups restricting the deprotonation at C_20_. This hypothesis was rationalized when attempts at quenching the supposed enolates with deuterated protic sources (D_2_O, AcOD) yielded no ^2^H incorporation at C_20_. 

Following this roadblock, we investigated the work of Boeckman et al., which probed the reactivity of cyclic enol ethers (as 3,4-dihydropyrans) by deprotonation using strong bases, followed by treatment with a wide range of electrophiles [24,25]. To the same extent, we aimed to prepare the equivalent enol ether (as 2,3-dihydrofuran) derivative of **5** to create the required reactive handle for the Suzuki–Miyaura reaction following these conditions. Heavy silyl protecting groups (TBS, TBDPS) were not considered to protect the C_3_ alcohol since studies have previously shown unpredictable migratory behavior in the required strongly basic conditions [26,27] and were not ideal in terms of mass economy. In contrast, the MOM-protecting group was chosen owing to its ability to withstand strong basic media, low molecular weight, and acid lability, enabling its removal during the acid-promoted spiroketalization whilst saving a deprotection step and enhancing the overall efficiency.

**Scheme 3:**
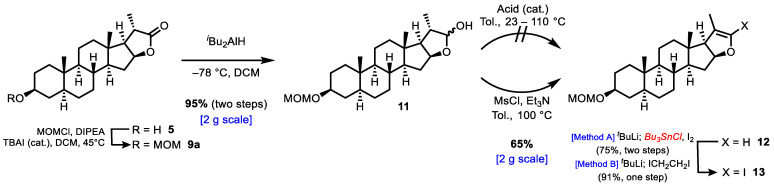
Preparation of iodo enol ether **13**.

We began our synthesis (Scheme 3) by treating **5** with MOMCl in the presence of DIPEA/TBAI [28] to afford MOM-protected lactone **9a**, followed by reduction with *^i^*Bu_2_AlH [20] to produce the corresponding hemiacetal **11** in 95% yield in two steps on 2 g scale as an inconsequential mix of diastereoisomers (2:1). Despite the screening of different catalytic acidic conditions previously used in dehydrations of hemiacetals [29], **12** could not be obtained cleanly without removing the MOM-protecting group. Fortunately, the activation of the hemiacetal using MsCl and subsequent elimination with an excess of Et_3_N at high temperatures [30] afforded the desired enol ether **12** in a modest 65% yield on a 2 g scale after passage through a short silica pad.

Next, we subjected **12** to similar conditions as those described by Boeckman [24,25]. Following lithiation, attempts at reacting various electrophilic halogen sources such as NBS, DBDMH, NCS, NIS, and I_2_ failed to produce the expected halogenated derivative. However, treatment of the organolithium intermediate with Bu_3_SnCl cleanly yielded the stannyl enol ether, which afforded the iodo enol ether **13** after reaction with molecular iodine (Scheme 3, Method A) [31]. Other interesting protocols have been reported for the synthesis of the equivalent obtained stannyl enol ether starting from a lactone derivative; however, they were too inefficient to be conducted on a large-scale [32,33]. Although the reaction with Bu_3_SnCl provided a direct route to **13**, the use of a tin intermediate is chemically inefficient, dangerous to handle on such scales, and traces of the toxic heavy metal would likely be carried to the final product used in in vivo and SAR studies. We circumvented this liability by reacting lithio-**12** with 1,2-diiodoethane (1,2-DIE) (Scheme 3, Method B), a niche electrophilic source of iodine. [34] To our delight, this methodology cleanly afforded **13** in 91% yield on a 2 g scale. A slight optimization of this protocol allowed us to use 3.0 eq. of both reagents (*^t^*BuLi and 1,2-DIE) instead of the original 4.0 eq. prescribed, while maintaining >97% conversion on gram scale. Further lowering of the equivalents (<3.0 eq.) led to poor conversions (<75%).

**Scheme 4:**
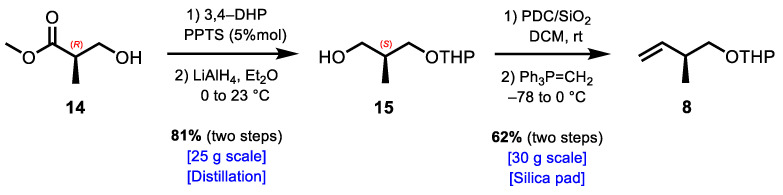
Scalable access to synthon **8**.

With the anchor of the Pd-mediated cross coupling installed on the steroidal scaffold, we then devised a scalable route to the desired olefin **8**. Commercially available (*R*)-Roche ester **14** was protected as THP followed by reduction using LiAlH_4_ to yield (*S*)-monoprotected diol **15** in 81% yield on a 25 g scale after distillation (Scheme 4) [35]. Subsequent oxidation using the Cornforth reagent (PDC) in conjunction with silica gel [36] cleanly yielded the corresponding aldehyde, which was then immediately subjected to a Wittig olefination [37]. The desired olefin **8** was cleanly obtained following a short silica pad in 62% yield after two steps on a 30 g scale.

Having obtained both coupling partners, we then explored the scalability of the reported one-pot transformation to obtain adduct **7** from **6** and **13**. Fortunately, we did not encounter any problems during the scale-up testing (up to 8 g) using the reported conditions [19]. Attempts at optimization by lowering the equivalents of alkylborane **6** (from 1.5 through to 1.1 eq.) and catalyst loading (20 mol% through to 1 mol%) were met with high amounts of reduction back to parent enol ether **12** (up to 20%), so the originally reported conditions were employed. The transformation sequence from **7** to the desired spirostanol **2** was then completed under strong mineral acidic conditions (methanolic HCl), as previous work highlighted the undesired isomerization at C_25_ when weaker mineral acids (e.g., AcOH) were used [38]. Spirostanol **2** was obtained in 8.1:1 d.r. at C_25_ in favor of the desired 25-(*S)* diastereoisomer in 74% yield on a 1 g scale after recrystallization. The observed imperfect diastereoselectivity could be attributed to the loss of optical purity of the intermediate aldehyde of **15** during the Wittig olefination through uncontrolled racemization under the strong basic conditions.

**Scheme 5:**
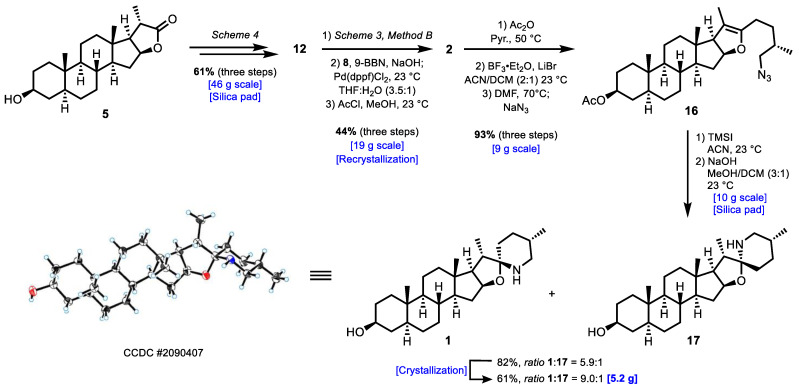
Streamlined chemical steps and ORTEP diagram of **1**.

After individually optimizing all the steps on a gram scale, we were able to streamline multiple steps to facilitate processing without compromising on either purity or yield (Scheme 5). We found that the protection, reduction, and dehydration sequence of **5** to **12** could be accomplished using crude intermediates in 65% yield on a 13 g scale and ultimately in 61% yield on a 46 g scale over three steps (avg. 85%/step), requiring only one silica pad after the third step. Minor decomposition products could be observed during the reduction step when the mixture warmed above −65 °C on the 13 g scale batch. In consequence, we decided to divide the 46 g scale batch in two equal parts to maintain better internal temperature control of the cryogenic mixture. The handling of the air-sensitive *^i^*Bu_2_AlH did not prove to be a safety hazard at this scale. Since the next reaction proved to be a safety hazard, we limited the scale of the transformation according to the *^t^*BuLi reagent bottle size and concentration. Transfer by canulation of a single reagent container (100 mL) of *^t^*BuLi to the reaction mixture limited our exposure to the pyrophoric reagent, which is important to avert potential accidents on this scale. [39] The iodination, cross-coupling, and spiroketalization steps from **12** to **2** were also streamlined to afford the desired spiroketal in 44% yield over three steps (avg. 76%/step) on a 19 g scale after recrystallization directly from the reaction mixture. 

With a large quantity of the desired spiroketal **2** in hand, we applied our previously used successful endgame sequence of transformations with no complications [15]. Compound **2** was acetylated using Ac_2_O in pyridine and then subjected to Lewis acid-mediated spiroketal opening using BF_3_·Et_2_O and LiBr, followed by substitution using NaN_3_ in DMF to yield the desired azide **16** in 93% yield over three steps (avg. 98%/step) and high purity (>99%) over three steps, requiring no purification. Subsequent reduction of the azide, followed by instant cyclization using TMSI (TMSCl/NaI) delivered the spiroaminoketal moiety, which, after ester hydrolysis (NaOH), produced the natural product **1** in a 5.9:1 ratio with the undesired 5,6-dihydrosolasodine **17**. The obtention of **17** as a minor product is a consequent of the unperfect diastereoselectivity in the obtention of **2** and the uncontrolled inversion of the C_25_ (S→R) during the TMSI-mediated reduction of **16** [40]. Purification by crystallization (vapor diffusion, see Supplementary Materials) generated crystals of tomatidine in a 9.0:1 ratio of **1**:**17** in 61% yield in two steps from azide **16** on a 10 g scale.

Overall, this sequence yielded 5.2 grams of tomatidine in a single batch, starting from 46 g of lactone **5** in 15.2% yield after 11 steps LLS (longest linear sequence). Another smaller-scale iteration of this sequence enabled us to produce a total of >7.5 grams of synthetic tomatidine in the laboratory. An analysis of a single crystal by X-ray diffraction (XRD) unequivocally confirmed the identity of **1** (Scheme 5) as its methanol co-crystal.

## 3. Experimental Section

### 3.1. General Remarks

Unless otherwise stated, all reactions were performed using commercial reagents bought from Sigma-Aldrich (Oakville, Canada), TCI America (Portland, OR, USA), Combi-Blocks (San Diego, CA, USA), Chem-Impex (Bensenville, IL, USA) and Strem Chemicals (Newburyport, MA, USA) without further purification and Teflon-coated stir bars. Reactions that required heating above room temperature (23 °C) were heated in an oil bath. Silica pads were performed using SiliaFlash™ P60 40 to 63 μm thick silica gel from Silicycle. Thin layer chromatography (TLC) was performed on glass-backed Silicycle SiliaPlate F-254 precoated plates with a thickness of 250 μm and a porosity of 60 Å. Compounds were visualized with UV light (254 nm), followed by staining with cerium ammonium molybdate (steroids) or potassium permanganate (nonsteroids) and heating. Nuclear magnetic resonance (NMR) spectra were obtained on a Bruker Ascend 400 spectrometer (Bruker, Ettlingen, Germany) and are reported in parts per million (ppm) for chemical shifts and hertz (Hz) for coupling constants (J). Residual chloroform (CHCl_3_) signals were used as an internal reference for the ^1^H (δ = 7.26 ppm) and ^13^C (δ = 77.16 ppm) spectra. The following abbreviations are used to describe the NMR multiplicities: br = broad, s = singlet, d = doublet, t = triplet, q = quartet, quint = quintet, sext = sextuplet, sept = septuplet, and m = multiplet. For the ^1^H NMR data of steroid intermediates, only assignable signals are listed. Infrared (FTIR) spectra were obtained on an ABB Bomem MB104 spectrophotometer (ABB Inc., Québec City, Canada) using a diamond-attenuated total reflectance (ATR) accessory. High-resolution mass spectra (HRMS) were obtained on a Nexera (Shimadzu) LC-QTOF mass spectrometer (Shimadzu Scientific Instruments, Columbia, MD, USA) coupled to a maXis (Bruker) mass spectrometer (ESI) using sodium formate as an internal standard. Melting points were measured on an Electrothermal MEL-TEMP 1101D melting point apparatus (Mettler-Toledo, Portland, OR, USA). XRD was performed on a KAPPA APEX-DUO (Bruker, Karlsruhe, Germany) diffractometer using a Cu radiation source.

### 3.2. Methods

#### 3.2.1. Synthesis of (2S)-2-Methyl-3-((tetrahydro-2H-pyran-2-yl)oxy)propan-1-ol (**15**)

A 500 mL round-bottom flask was charged with a stir bar, DCM (200 mL), (*R*)-Roche ester **14** (24.9 g, 211 mmol, 1.0 eq.), and 3,4-dihydro-2*H*-pyran (26.9 mL, 295 mmol, 1.4 eq.). Pyridinium *p*-toluenesulfonate (2.65 g, 10.5 mmol, 0.05 eq.) was then added, and the mixture was stirred at room temperature. After 5 h, the reaction was diluted with DCM (200 mL), and the organic phase was washed with water (50 mL). The organic phase was collected, and the aqueous phase was extracted with DCM (3 × 50 mL). The combined organic layers were washed with brine (25 mL), dried over Na_2_SO_4_, and concentrated *in vacuo* to yield the crude THP ester as a colorless oil.

A 1 L, two-neck round-bottom flask was charged with Et_2_O (175 mL) and lithium aluminum hydride (8.00 g, 211 mmol, 1.0 eq.). A thermometer was fitted, and the suspension was vigorously stirred and cooled to 0 °C using an ice bath. To this suspension was added a solution of the THP ester prepared above in Et_2_O (200 mL + 50 mL wash) via canulation at a slow rate that kept the internal temperature below 15 °C. After the canulation was complete, the mixture was warmed to room temperature and stirred overnight. The mixture was then cooled back to 0 °C and quenched by careful addition of sodium sulfate decahydrate (50.9 g, 158 mmol, 0.75 eq.). The suspension was warmed to room temperature and stirred vigorously for 1 h. Subsequently, this suspension was filtered using a Büchner funnel and concentrated *in vacuo*. The obtained residue was distilled under reduced pressure to yield monoprotected diol **15** (29.6 g, 81% over two steps) as a colorless oil.

^1^H NMR (400 MHz, CDCl_3_) δ (ppm) 4.55 (t, 1H, *J* = 4.4 Hz), 3.86–3.79 (m, 1H), 3.80 (q, 0.5H, *J* = 4.8 Hz), 3.64 (dd, 1H, *J* = 9.6, 8.4 Hz), 3.59–3.54 (m, 1.5H), 3.53–3.47 (m, 1H), 3.44 (q, 0.5H, *J* = 4.4 Hz), 3.34 (dd, 0.5H, *J* = 9.2, 5.2 Hz), 2.79–2.73 (m, 1H), 2.05–1.95 (m, 1H), 1.80–1.64 (m, 2H), 1.58–1.46 (m, 4H), 0.89 (dd, 3H, *J* = 6.8, 4.4 Hz). ^13^C NMR (100 MHz, CDCl_3_) δ (ppm) 99.4, 99.2, 72.1, 72.0, 67.3, 67.2, 62.6, 62.5, 35.8, 35.5, 30.7, 30.6, 25.4, 25.3, 19.7, 13.7, 13.6. HRMS-ESI (*m/z*) calcd for C_9_H_18_O_3_: 197.1148 [M + Na]^+^ found: 197.1151 [M + Na]^+^. FTIR-ATR (neat) υ [cm^−1^] 3436, 2941, 2871, 1739, 1120, 1020, 973, 900, 867, 813. R_f_ 0.45 (50% EtOAc/hexanes) [KMnO_4_]. Bp 135–140 °C (30 mmHg).

#### 3.2.2. Synthesis of 2-(((S)-2-Methylbut-3-en-1-yl)oxy)tetrahydro-2H-pyran (**8**)

A mixture of pyridinium dichromate (75.6 g, 201 mmol, 2.0 eq.) and silica gel (75.6 g) was ground to a fine powder using a mortar and pestle. This homogenous solid was then transferred to a 1 L round-bottom flask containing DCM (200 mL) and vigorously stirred using a large stir bar. A solution of **15** (17.5 g, 100 mmol, 1.0 eq.) in DCM (60 mL) was then added dropwise over 15 min. This suspension was stirred at room temperature for 48 h. The mixture was diluted with Et_2_O (500 mL) and then filtered through a 2:1 silica gel/Celite™ pad. This mixture was subsequently concentrated *in vacuo* and coevaporated using tetrahydrofuran (THF, 3 × 75 mL) to obtain the corresponding aldehyde. In another 1 L round-bottom flask, a *^n^*BuLi solution (2.50 M in hexanes, 100 mL, 251 mmol, 2.5 eq.) was canulated into a suspension of methyltriphenylphosphonium bromide (93.3 g, 261 mmol, 2.6 eq.) in anhydrous THF (415 mL) at 0 °C. After stirring for 30 min, this solution was cooled to −78 °C and a solution of the crude aldehyde in THF (100 mL + 50 mL wash) was transferred via canulation. After 30 min at this temperature, the mixture was warmed to 0 °C and stirred for further 45 min. The mixture was cooled back to −78 °C and then quenched with sat. NH_4_Cl_(aq.)_ (150 mL). The mixture was slowly warmed to room temperature over 1 h and then transferred to an extraction funnel containing water (250 mL). The organic layer was collected, and the aqueous layer was extracted with EtOAc (3 × 200 mL). The combined organic phases were washed with brine (100 mL), dried over Na_2_SO_4_, and concentrated *in vacuo* to produce a light-orange oil. The crude product was passed through a silica pad (6 cm × 10 cm) using 20% EtOAc/hexanes (1 L) as an eluent. The filtrate was concentrated *in vacuo* to yield olefin **8** (10.6 g, 62%, >95% purity by NMR) as a light-yellow oil, which was used in the next step without further purification.

^1^H NMR (400 MHz, CDCl_3_) δ (ppm) 5.84–5.74 (m, 1H), 5.04 (dd, 1H, *J* = 8.8, 1.2 Hz), 4.98 (dd, 1H, *J* = 5.2, 1.2 Hz), 4.57 (t, 1H, *J* = 3.6 Hz), 3.87–3.81 (m, 1H), 3.58 (ddd, 1H, *J* = 36.4, 9.6, 6.8 Hz), 3.50–3.45 (m, 1H), 2.46 (quint, 1H, *J* = 6.8 Hz), 1.85–1.76 (m, 1H), 1.73–1.64 (m, 1H), 1.60–1.47 (m, 4H), 1.02 (dd, 3H, *J* = 6.8, 4.4 Hz). ^13^C NMR (100 MHz, CDCl_3_) δ (ppm) 141.5, 141.4, 114.1, 114.0, 98.9, 98.8, 72.2, 72.1, 62.2, 62.1, 37.9, 37.8, 30.7, 25.6, 19.6, 16.8, 16.7. HRMS-ESI (*m/z*) calcd for C_10_H_18_O_2_: 193.1200 [M + Na]^+^ found: 193.1203 [M + Na]^+^. FTIR-ATR (neat) υ [cm^−1^] 2925, 2869, 1456, 1201, 1120, 1031, 973, 904, 869, 815. R_f_ 0.50 (5% EtOAc/hexanes) [KMnO_4_].

#### 3.2.3. Synthesis of (2aS,4S,6aS,6bS,8aS,8bR,9S,11aS,12aS,12bR)-4-(Methoxymethoxy)-6a,8a,9-trimethyloctadecahydro-10H-naphtho[2′,1′:4,5]indeno[2,1-b]furan-10-one (**9a**)

Dinorcholanic lactone **5** was prepared from commercial diosgenin (Chem Impex, Catalog No. 24131) by hydrogenation [41], B-V oxidation [20], and recrystallization from EtOAc. A 1 L, two-neck round-bottom flask was charged with a stir bar, **5** (43.7 g, 126 mmol, 1.0 eq.), tetrabutylammonium iodide (3.26 g, 8.82 mmol, 0.07 eq.), and DCM (375 mL). N,N-Diisopropylethylamine (30.7 mL, 176 mmol, 1.4 eq.) was then added in one portion. A condenser and an addition funnel containing chloromethyl methyl ether (11.5 mL, 151 mmol, 1.2 eq.) in DCM (50 mL) were connected. The mixture was stirred for 10 min under a flow of Ar, and the MOMCl solution was then added dropwise over 10 min. Subsequently, the reaction mixture was heated to reflux. After 3 h, the mixture was cooled down to room temperature, transferred to an extraction funnel, and washed with 1N HCl_(aq.)_ (150 mL)*. The organic phase was collected, and the aqueous phase was extracted with DCM (3 × 100 mL). The organic layers were combined, washed with brine (50 mL), dried over Na_2_SO_4_, and then concentrated *in vacuo* to yield MOM lactone **9a** (49.1 g, >99% purity by NMR) as a light-brown solid, which was used in the next step without purification.

*NOTE: Vigorous gas build-up, vent frequently.

^1^H NMR (400 MHz, CDCl_3_) δ (ppm) 4.92 (dt, 1H, *J* = 4.8, 7.6 Hz), 4.66 (s, 2H), 3.47 (sept, 1H, *J* = 4.8 Hz), 3.35 (s, 3H), 2.56 (q, 1H, *J* = 7.6 Hz), 2.25 (quint, 1H, *J* = 7.2 Hz), 1.84 (d, 1H, *J* = 7.6 Hz), 1.30 (d, 3H, *J* = 7.6 Hz), 0.81 (s, 3H), 0.73 (s, 3H). ^13^C NMR (100 MHz, CDCl_3_) δ (ppm) 181.5, 94.7, 82.9, 76.3, 59.2, 55.3, 54.7, 54.5, 44.9, 41.9, 38.5, 37.1, 36.2, 35.9, 35.3, 34.9, 33.1, 32.3, 28.8, 28.6, 20.6, 18.1, 14.0, 12.4. HRMS-ESI (*m/z*) calcd for C_24_H_38_O_4_: 413.2662 [M + Na]^+^ found: 413.2660 [M + Na]^+^. FTIR-ATR (neat) υ [cm^−1^] 2927, 2873, 2844, 1755, 1454, 1188, 1105, 1029, 879, 671, 628. R_f_ 0.45 (20% EtOAc/hexanes) [CAM]. Mp 143–145 °C.

#### 3.2.4. Synthesis of (2aS,4S,6aS,6bS,8aS,8bR,9S,11aS,12aS,12bR)-4-(Methoxymethoxy)-6a,8a,9-trimethyloctadecahydro-1H-naphtho[2′,1′:4,5]indeno[2,1-b]furan-10-ol (**11**)

Two 1 L, two-neck round-bottom flasks were each fitted with a stir bar and a thermometer. The flasks were then charged with crude **9a** (23.2 g, 59.5 mmol, 1.0 eq.) and purged of air and moisture by alternately applying vacuum and Ar_(g)_ in combination with torching (three cycles). Anhydrous DCM (240 mL) was then added and the mixture was cooled to −78 °C using a dry ice/acetone bath. A diisobutylaluminium hydride solution (1 M in DCM, 71.4 mL, 71.4 mmol, 1.2 eq.) was then added dropwise to each flask at a slow rate to ensure the temperature was maintained below −70 °C. Once the addition was complete, the resultant solutions were further stirred for 15 min at this temperature and subsequently quenched by dropwise addition of EtOH (8 mL). The mixtures were allowed to warm to room temperature and sat. sodium potassium tartrate tetrahydrate_(aq.)_ (150 mL) was added, followed by vigorously stirring overnight. The mixtures were then combined in an extraction funnel containing water (300 mL). The organic layer was collected, and the aqueous layer was extracted with DCM (4 × 150 mL). The combined organic phases were washed with brine (100 mL), dried over Na_2_SO_4_, and then concentrated *in vacuo* to yield MOM hemiacetal **11** (46.4 g, 90–92% purity by NMR) as a light-brown foam in an inconsequential 2:1 mixture of diastereoisomers, which was used directly in the next step without purification. A small sample (30 mg) was purified by preparative TLC to collect analytical data.

^1^H NMR (400 MHz, CDCl_3_) δ (ppm) 5.46–5.45 (d, 0.66H, 22-H_maj_, *J* = 3.6 Hz), 4.87 (t, 0.33H, 22-H_min_, *J* = 5.6 Hz), 4.72–4.65 (m, 0.66H, 16-H_maj_), 4.67 (s, 2H), 4.35 (dt, 0.33H, 16-H_min_, *J* = 6.0, 8.0 Hz), 3.48 (sept. 1H, *J* = 6.4 Hz), 3.36 (s, 3H), 3.00 (br s, 0.32H, 22-O*H_min_*), 2.45 (br s, 0.66H, 22-O*H*_maj_), 2.26 (sext, 0.66H, 20-H_maj_, *J* = 7.2 Hz), 2.06–1.97 (m, 1H), 1.90 (sext, 0.33H, 20-H_min_, *J* = 6.8 Hz), 1.09 (d, 1H, H_min_-C_21_ , *J* = 7.2 Hz), 1.04 (d, 2H, H_maj_-C_21_, *J* = 7.2 Hz), 0.87 (s, 1H, H_min_-C_18_), 0.82 (s, 1H, H_min_-C_19_), 0.81 (s, 2H, H_maj_-C_18_), 0.76 (s, 2H, H_maj_-C_19_). ^13^C NMR (100 MHz, CDCl_3_) δ (ppm) 108.4, 103.3, 94.6, 82.3, 80.4, 76.3, 62.7, 62.1, 56.4, 56.1, 55.2, 54.5, 54.4, 44.9, 41.3, 40.9, 39.9, 39.8, 37.1, 37.08, 37.06, 35.8, 35.3, 35.2, 35.1, 32.7, 32.3, 32.2, 28.7, 21.0, 20.9, 18.2, 16.7, 16.2, 14.9, 12.4, 12.3. HRMS-ESI (*m/z*) calcd for C_24_H_40_O_4_: 415.2819 [M + Na]^+^ found: 415.2811 [M + Na]^+^. FTIR-ATR (neat) υ [cm^−1^] 3388, 2927, 2846, 1448, 1379, 1143, 1105, 1039, 985, 929. R_f_ 0.30 (20% EtOAc/hexanes) [CAM]. Mp 133–135 °C.

#### 3.2.5. Synthesis of (2aS,4S,6aS,6bS,8aS,8bS,11aS,12aS,12bR)-4-(Methoxymethoxy)-6a,8a,9-trimethyl 2,2a,3,4,5,6,6a,6b,7,8,8a,8b,11a,12,12a, 12b-hexadecahydro-1H-naphtho[2′,1′:4,5]indeno[2,1-b]furan (**12**)

A 1 L, two-neck round-bottom flask was charged with a stir bar and crude **11** (46.4 g, 119 mmol, 1.0 eq.). Toluene (475 mL) and freshly distilled triethylamine (133 mL, 954 mmol, 8.0 eq.) were added, and the flask was fitted with a reflux condenser and an addition funnel containing freshly distilled methanesulfonyl chloride (36.9 mL, 477 mmol, 4.0 eq.). The flask was then stirred under a flow of Ar_(g)_ for 10 min, followed by dropwise addition of MsCl over 15 min*. After the addition was complete, the mixture was stirred at 100 °C for 16 h. The mixture was then cooled to room temperature, followed by a second addition of triethylamine (33.2 mL, 238 mmol, 2.0 eq) and methanesulfonyl chloride (9.23 mL, 119 mmol, 1.0 eq.). This mixture was then further stirred for 3 h at 100 °C. The mixture was then cooled to room temperature, quenched with sat. NH_4_Cl_(aq.)_ (200 mL), and vigorously stirred for 30 min. The mixture was subsequently transferred to an extraction funnel containing water (400 mL) and extracted with EtOAc (4 × 300 mL). The organic layers were combined and washed with brine (100 mL), dried over Na_2_SO_4_, and then concentrated *in vacuo* to yield a dark brown oil, which was passed through a silica pad (6 cm × 14 cm) using 10% EtOAc/hexanes (2 L) as an eluent. The filtrate was then concentrated *in vacuo* to yield enol ether **12** (27.3 g, 61%, three steps) as an off-white solid.

*NOTE: Strong exothermic reaction.

^1^H NMR (400 MHz, CDCl_3_) δ (ppm) 6.00 (s, 1H), 4.88–4.82 (m, 1H), 4.68 (s, 2H), 3.49 (sept, 1H, *J* = 4.8 Hz), 3.36 (s, 3H), 2.44 (d, 1H, *J* = 10.4 Hz), 2.20–2.13 (m, 1H), 1.61 (s, 3H), 0.82 (s, 3H), 0.71 (s, 3H). ^13^C NMR (100 MHz, CDCl_3_) δ (ppm) 141.0, 111.1, 94.7, 87.3, 76.4, 63.0, 55.3, 54.9, 54.5, 45.0, 43.3, 39.8, 37.2, 35.9, 35.4, 35.0, 34.2, 32.6, 28.9, 28.8, 21.3, 14.3, 12.4, 11.5. HRMS-ESI (*m/z*) calcd for C_24_H_38_O_3_: 397.2713 [M + Na]^+^ found: 397.2708 [M+Na] ^+^ . FTIR-ATR (neat) υ [cm^−1^] 2935, 2879, 1144, 1379, 1103, 1085, 1045, 931, 914, 848, 617. R_f_ 0.50 (10% EtOAc/hexanes) [CAM]. Mp 120–122 °C.

#### 3.2.6. Synthesis of (2aS,4S,6aS,6bS,8aS,8bS,11aS,12aS,12bR)-10-Iodo-4-(methoxymethoxy)-6a,8a,9-trimethyl-2,2a,3,4,5,6,6a,6b,7,8,8a,8b,11a, 12,12a,12b-hexadecahydro-1H-naphtho[2′,1′:4,5]indeno[2,1-b]furan (**13**)

A 1 L round-bottom flask was charged with **12** (18.8 g, 50.2 mmol, 1.0 eq.) and a stir bar*. The flask was purged of air and moisture by alternately applying vacuum and Ar_(g)_ (three cycles) in combination with torching. Anhydrous THF (100 mL) was then added, and the mixture was cooled to −78 °C in a dry ice/acetone bath. Freshly titrated [42] *^t^*BuLi (1.50 M in pentane, 100 mL, 151 mmol, 3.0 eq.) was then transferred to the chilled flask via canulation over 15 min. This mixture was stirred for 5 min at −78 °C and then warmed to 0 °C using an ice bath. After 30 min at this temperature, the flask was placed back in the dry ice/acetone bath and cooled to −78 °C.

Under Ar atmosphere, a 250 mL round-bottom flask was charged with freshly purified [43] 1,2-diiodoethane (42.5 g, 151 mmol, 3.0 eq.) and dissolved in anhydrous THF (100 mL + 10 mL wash). This solution was then added to the lithiated enol ether flask by canulation over 15 min. After the addition, the cooling bath was replaced with a room temperature water bath, and the reaction was allowed to proceed for 1 h as the mixture slowly developed a deep purple color. Sat. Na_2_S_2_O_3(aq)_ (250 mL) was then added to the flask and stirred vigorously. After 15 min, this mixture was transferred to an extraction funnel containing water (300 mL). The organic layer was collected, and the aqueous layer was extracted with EtOAc (3 × 200 mL). The organic layers were combined, washed with brine (75 mL), dried over Na_2_SO_4_, and concentrated *in vacuo*. The product was passed through a silica pad (6 cm × 12 cm) using 15% EtOAc/hexanes (2 L) as an eluent to yield iodo enol ether **13** (24.7 g) as a light-yellow solid following concentration, which was used immediately in the next step. NMR analysis of the crude mixture showed 99% conversion of the starting material [44].

*NOTE: **13** was shielded from light at all times. **13** was found to be unstable even when stored under Ar_(g)_ in a dark cold freezer after one week and needs to be consumed directly after it is synthesized. After being stored for 24 h in CDCl_3_ in an NMR tube at room temperature, **13** underwent decomposition, and the solution in the tube turned to a dark purple color.

^1^H NMR (400 MHz, CDCl_3_) δ (ppm) 4.97–4.91 (m, 1H), 4.65 (s, 2H), 3.47 (sept, 1H, *J* = 4.8 Hz), 3.34 (s, 3H), 2.43 (d, 1H, *J* = 10.0 Hz), 2.18 (quint, 1H, *J* = 6.0 Hz), 1.63 (s, 3H), 0.80 (s, 3H), 0.66 (s, 3H). ^13^C NMR (100 MHz, CDCl_3_) δ (ppm) 117.3, 100.0, 94.3, 88.5, 76.2, 62.4, 55.2, 54.3, 54.2, 44.9, 44.0, 39.4, 37.1, 35.8, 35.3, 34.9, 32.5, 28.8, 28.7, 21.2, 14.6, 14.3, 12.4 HRMS-ESI (*m/z*) calcd for C_24_H_37_O_7_I: 501.1860 [M+H]^+^ found: 501.1853 [M+H]^+^. FTIR-ATR (neat) υ [cm^−1^] 2925, 2850, 1446, 1384, 1107, 1047, 1033, 906, 833, 725, 619. R_f_ 0.48 (10% EtOAc/hexanes) [UV, CAM]. Mp 123–125 °C.

#### 3.2.7. Synthesis of (2aS,4S,6aS,6bS,8aS,8bS,11aS,12aS,12bR)-4-(Methoxymethoxy)-6a,8a,9-trimethyl-10-((3S)-3-methyl-4-((tetrahydro-2H-pyran-2-yl)oxy)butyl)-2,2a,3,4,5,6,6a,6b,7,8,8a,8b,11a,12,12a,12b-hexadecahydro-1H-naphtho[2′,1′:4,5]indeno[2,1-b]furan (**7**)

A 1 L round-bottom flask was charged with **8** (12.8 g, 75.2 mmol, 1.5 eq.) and a stir bar. The flask was purged of air and moisture by alternately applying vacuum and Ar_(g)_ (three cycles), and anhydrous THF (300 mL) was then added. A 9-borabicyclo[3.3.1]nonane solution (0.5 M in THF, 300 mL, 150 mmol, 3.0 eq.) was added dropwise over 30 min. The resulting mixture was stirred for 3 h at room temperature. Then, NaOH_(aq.)_ (1 M, 150 mL, 150 mmol, 3.0 eq.) was added carefully, and the mixture was stirred for a further 30 min at room temperature to prepare intermediate alkylborane **6**.

Meanwhile, a 3 L flask was charged with crude **13** and a stir bar, followed by a 3:1 THF/H_2_O mixture (500 mL). [1,1′-Bis(diphenyl-phosphino)ferrocene]dichloropalladium (II) complex with dichloromethane (8.19 g, 10.0 mmol, 0.2 eq.) was added in one portion and then stirred under Ar_(g)_ for 10 min. The hydroboration reaction mixture was then canulated to the main reaction over 15 min in a final THF/H_2_O ratio of 3.5:1. After the transfer was complete, the mixture, which turned from bright red to dark brown upon addition of the alkylborane solution, was stirred at room temperature. After 5 h, a TLC analysis of the reaction showed full consumption of **13**. Hexanes (800 mL) were then added, and the resulting suspension was filtered through a 1:1 Celite™/silica gel pad, which was further washed with 20% EtOAc/hexanes (300 mL). The filtrates were combined and added to an extraction funnel containing water (600 mL). The organic layer was collected, and the aqueous layer was extracted with EtOAc (1 × 200 mL). The organic layers were combined, washed with brine (150 mL), dried over Na_2_SO_4_, and concentrated *in vacuo* to obtain a brown oil. The crude material was passed through a silica pad (6 cm × 12 cm) using 20% EtOAc/hexanes (1.5 L) as an eluent. The filtrate was concentrated *in vacuo* to yield adduct **7** (26.1 g) as a light-brown pasty solid, which was used immediately in the next step. A small sample (35 mg) was purified by preparative TLC to collect analytical data.

^1^H NMR (400 MHz, CDCl_3_) δ (ppm) 4.73–4.64 (m, 1H), 4.66 (s, 2H), 4.55 (s, 1H), 3.87–3.81 (m, 1H), 3.95 (dd, 0.5H, *J* = 9.6, 6.0 Hz), 3.52–3.43 (m, 2.5H), 3.22 (dd, 0.5H, *J* = 9.2, 5.6 Hz), 3.13 (dd, 0.5H, *J* = 9.6, 6.8 Hz), 2.43 (d, 1H, *J* = 10.0 Hz), 2.16–2.05 (m, 3H), 1.56 (s, 3H), 0.93 (dd, 3H, *J* = 6.8, 5.2 Hz), 0.81 (s, 3H), 0.65 (s, 3H). ^13^C NMR (100 MHz, CDCl_3_) δ (ppm) 152.0, 151.9, 103.6, 103.6, 99.1, 98.8, 94.6, 84.4, 76.4, 72.9, 72.8, 64.5, 62.2, 62.1, 55.2, 54.9, 54.5, 44.9, 43.7, 39.9, 37.2, 35.8, 35.4, 35.0, 34.2, 33.3, 33.2, 32.6, 32.2, 31.3, 30.8, 28.8, 26.4, 25.7, 23.6, 23.5, 22.1, 21.3, 19.6, 17.2, 17.1, 14.3, 12.4, 11.8. HRMS-ESI (*m/z*) calcd for C_34_H_56_O_5_: 567.4020 [M+Na]^+^ found: 567.4015 [M+Na]^+^. FTIR-ATR (neat) υ [cm^−1^] 2925, 2848, 1448, 1380, 1107, 1033, 975, 904, 867, 815. R_f_ 0.65 (15% EtOAc/hexanes). Mp 78–80 °C.

#### 3.2.8. Synthesis of (3β,5α,25S)-Spirostan-3-ol (**2**) 

A 1 L round-bottom flask was charged with crude **7** and a stir bar, followed by addition of MeOH (390 mL). The solution was adjusted to pH 1 by careful addition of acetyl chloride (5.34 mL, 75.2 mmol, 1.5 eq.), and the resulting mixture was stirred at room temperature. After 30 h, a thick white precipitate was formed, and an NMR analysis of the crude mixture showed full conversion of the starting material and its intermediates. The crude mixture was then transferred to an Erlenmeyer, recrystallized directly from the reaction mixture, and filtered through a Büchner funnel to yield **2** (9.15 g, 44%, three steps) as a white solid with an 8.1:1 d.r. at C_25_*.

*NOTE: Only the major C_25_ isomer was characterized. The diastereoisomeric ratio was calculated by integrating **2** (d, δ = 3.29 ppm) and 25-*epi*-**2** (t, δ = 3.37 ppm).^38^ See Supplementary Materials. 

^1^H NMR (400 MHz, CDCl_3_) δ (ppm) 4.39 (q, 1H, *J* = 7.2 Hz), 3.94 (dd, 1H, *J* = 10.8, 2.4 Hz), 3.58 (sept, 1H, *J* = 4.8 Hz), 3.29 (d, 1H, *J* = 11.2 Hz), 1.07 (d, 3H, *J* = 7.2 Hz), 0.98 (d, 3H, *J* = 6.8 Hz), 0.81 (s, 3H), 0.75 (s, 3H). ^13^C NMR (100 MHz, CDCl_3_) δ (ppm) 109.7, 81.1, 71.4, 65.3, 62.2, 56.4, 54.5, 44.9, 42.3, 40.7, 40.2, 38.3, 37.1, 35.7, 35.3, 32.4, 31.7, 31.6, 28.7, 27.2, 26.1, 25.9, 21.2, 16.6, 16.2, 14.5, 12.5. HRMS-ESI (*m/z*) calcd for C_27_H_44_O_3_: 439.3183 [M+Na]^+^ found: 439.3173 [M+Na]^+^. FTIR-ATR (neat) υ [cm^−^^1^] 3523, 3386, 2929, 2846, 1448, 1369, 1172, 1047, 981, 918, 850. R_f_ 0.50 (30% EtOAc/hexanes) [CAM]. Mp 194–197 °C [lit. 197–203 °C] [45]. The spectroscopic data were in agreement with previously reported data [38].

#### 3.2.9. Synthesis of (2aS,4S,6aS,6bS,8aS,8bS,11aS,12aS,12bR)-10-((S)-4-Azido-3-methylbutyl)-6a,8a,9-trimethyl-2,2a,3,4,5,6,6a,6b,7,8,8a,8b,11a,12,12a,12b-hexadecahydro-1H-naphtho[2′,1′:4,5]indeno[2,1-b]furan-4-yl acetate (**16**) 

A 250 mL round-bottom flask was charged with **2** (9.15 g, 27.8 mmol, 1.0 eq.) and pyridine (87 mL). This mixture was warmed to 50 °C, and acetic anhydride (16.6 mL, 176 mmol, 8.0 eq.) was then added in one portion. After 2 h at this temperature, the mixture was poured into a 1 L Erlenmeyer containing ice-cold water (500 mL) and a stir bar. This suspension was vigorously stirred for 15 min and filtered using a Büchner funnel. The resulting pasty solid was then collected and dried under high vacuum overnight to yield OAc-**2** (10.1 g) as a white fluffy solid.

A 1 L, two-neck round-bottom flask was charged with crude OAc-**2**, lithium bromide (19.1 g, 220 mmol, 10.0 eq.), and a stir bar. This flask was purged of air and moisture by alternately applying vacuum and Ar_(g)_ (three cycles)*. DCM (190 mL) and anhydrous acetonitrile (95 mL) were then added, and the mixture was stirred vigorously before adding BF_3_·Et_2_O (27.2 mL, 220 mmol, 10.0 eq.) dropwise over 10 min at room temperature. After 3 h, sat. NaHCO_3(aq.)_ (50 mL) was slowly added, followed by water (200 mL). This mixture was transferred to an extraction funnel, the organic layer was collected, and the aqueous layer was extracted with DCM (3 × 100 mL). The organic layers were combined, washed with brine (40 mL), dried over Na_2_SO_4_, and concentrated *in vacuo*. The crude mixture was then transferred to a 250 mL round-bottom flask, dissolved in DMF (95 mL), and heated to 70 °C. After stirring for 2 h at this temperature, sodium azide (4.29 g, 66.1 mmol, 3.0 eq.) was added, and the mixture was stirred for further 3 h at 70 °C. Subsequently, the mixture was cooled to room temperature, transferred to an extraction funnel containing water (300 mL), and extracted with DCM (4 × 100 mL). The organic layers were combined, washed with brine (100 mL), dried over Na_2_SO_4_, and then concentrated *in vacuo* to yield **16** (9.91 g, 93% over three steps, >99% purity by NMR) as a beige waxy solid. This material was used directly in the next step without purification.

*NOTE: Glass vacuum adaptors were used to keep the mixture under Ar_(g)_ during the reaction—BF_3_·Et_2_O corroded and clogged regular needles.

OAc-**2**: ^1^H NMR* (400 MHz, CDCl_3_) δ (ppm) 4.68 (sept, 1H, *J* = 4.8 Hz), 4.39 (q, 1H, *J* = 7.2 Hz), 3.94 (dd, 1H, *J* = 10.8, 2.8 Hz), 3.29 (d, 1H, *J* = 11.2 Hz), 2.01 (s, 3H), 1.07 (d, 3H, *J* = 7.2 Hz), 0.98 (d, 3H, *J* = 6.8 Hz), 0.83 (s, 3H), 0.75 (s, 3H). ^13^C NMR (100 MHz, CDCl_3_) δ (ppm) 170.9, 109.9, 81.1, 77.4, 73.8, 65.3, 62.1, 56.4, 54.3, 44.8, 42.3, 40.7, 40.2, 36.9, 35.7, 35.2, 34.2, 32.3, 31.9, 28.7, 27.6, 27.2, 26.1, 25.9, 21.6, 21.1, 16.7, 16.2, 14.5, 12.4. HRMS-ESI (*m/z*) calcd for C_29_H_46_O_4_: 481.3288 [M + Na]^+^ found: 481.3288 [M + Na]^+^. FTIR-ATR (neat) υ [cm^−1^] 2945, 2846, 1724, 1446, 1379, 1240, 1027, 987, 919, 854, 611. R_f_ 0.50 (10% EtOAc/hexanes) [CAM]. Mp 172–174 °C [lit. 176–178 °C] [46].

*NOTE: Only the major C_25_ isomer was characterized. The ratio of OAc-**2** to 25-*epi*-OAc-**2** was identical to the ratio of **2** to 25-*epi*-**2**.

**16**: ^1^H NMR (400 MHz, CDCl_3_) δ (ppm) 4.74–4.63 (m, 2H), 3.21 (dd, 1H, *J* = 12.0, 5.6 Hz), 3.07 (dd, 1H, *J* = 12.0, 7.2 Hz), 2.44 (d, 1H, *J* = 10.0 Hz), 2.17–2.07 (m, 3H), 2.00 (s, 3H), 0.95 (d, 3H, *J* = 6.8 Hz), 0.82 (s, 3H), 0.65 (s, 3H). ^13^C NMR (100 MHz, CDCl_3_) δ (ppm) 170.8, 151.3, 104.2, 84.5, 73.8, 64.4, 57.8, 54.8, 54.3, 44.7, 43.7, 39.8, 36.9, 35.7, 34.9, 34.1, 33.2, 32.4, 31.5, 28.6, 27.6, 23.3, 21.6, 21.3, 17.6, 14.3, 12.4, 11.8. HRMS-ESI (*m/z*) calcd for C_27_H_42_N_3_O: 484.3534 [M + H]^+^ found: 484.3528 [M + H]^+^. FTIR-ATR (neat) υ [cm^−1^] 2916, 2844, 2096, 1735, 1689, 1448, 1367, 1236, 1027, 663. R_f_ 0.52 (10% EtOAc/hexanes) [CAM]. Mp 58–60 °C

#### 3.2.10. Synthesis of Tomatidine (**1**)

A 1 L round-bottom flask was charged with **16** (9.91 g, 20.5 mmol, 1.0 eq.), sodium iodide (6.76 g, 45.1 mmol, 2.2 eq.), and a stir bar, followed by addition of ACN (240 mL). This mixture was stirred until all the solids were dissolved (30 min) before a solution of trimethylsilyl chloride (5.98 mL, 47.1 mmol, 2.3 eq.) in ACN (40 mL) was added dropwise. The mixture was stirred at room temperature and quickly turned purple. After stirring for 1 h at room temperature, sat. Na_2_S_2_O_3(aq.)_ (60 mL) was added, and the pH was adjusted to 7 using 2 M NaOH (ca. 40 mL). The volatiles were then removed *in vacuo* to give a yellow suspension, which was added to water (100 mL) and extracted with CHCl_3_ (4 × 75 mL). The organic layers were combined, washed with brine (30 mL), dried over Na_2_SO_4_, and concentrated *in vacuo*. The obtained solid was then transferred to a 1 L round-bottom flask and dissolved in a 3:1 MeOH/DCM mixture (350 mL). Subsequently, a 3 M NaOH solution (34.1 mL, 102 mmol, 5.0 eq.) was added, and the reaction was stirred at room temperature. After 2 h, 1 M HCl (ca. 70 mL) was used to adjust the mixture to pH 6. The volatiles were removed *in vacuo*, and the mixture was suspended in water (150 mL) and extracted with CHCl_3_ (4 × 75 mL). The organic layers were combined, washed with brine (30 mL), dried over Na_2_SO_4_, and concentrated *in vacuo*. The crude material was passed through a silica pad (6 cm × 14 cm) using 60:39:1 EtOAc/hexanes:Et_3_N (1.5 L) as an eluent. The filtrate was concentrated *in vacuo* to yield an amorphous yellow solid (6.95 g, 82%) in a 5.9:1 ratio of **1** to **17**. Product **1** was crystallized selectively by dissolving the crude solid in a 3:1 MeOH/DCM mixture (80 mL) in a crystallizing dish, which was placed in a hermetically closed larger container containing MeOH (See Supplementary Materials). After 48 h, the supernatant was removed from the crystallizing dish, and the crystals were collected and dried. This process was repeated a second time, and the products were combined to yield **1** (5.17 g, 61%, two steps) as colorless crystals in a 9:1 ratio of **1** to **17** (purity 90%).

^1^H NMR (400 MHz, CDCl_3_) δ (ppm) 4.10 (q, 1H, *J* = 8.4 Hz), 3.55 (sept, 1H, *J* = 4.8 Hz), 2.75–2.69 (m, 2H), 2.00–1.94 (m, 1H), 0.94 (d, 3H, *J* = 7.2 Hz), 0.83 (d, 3H, *J* = 6.4 Hz), 0.80 (s, 3H), 0.79 (s, 3H). ^13^C NMR (100 MHz, CDCl_3_) δ (ppm) 99.1, 78.6, 71.3, 62.1, 55.9, 54.5, 50.3, 44.9, 43.1, 40.9, 40.3, 38.3, 37.1, 35.7, 35.2, 32.8, 32.4, 31.6, 31.1, 28.7, 28.6, 26.7, 21.2, 19.5, 17.1, 15.9, 12.5. HRMS-ESI (*m/z*) calcd for C_27_H_45_NO_2_: 416.3523 [M+H]^+^ found: 416.3517 [M+H]^+^. FTIR-ATR (neat) υ [cm^−^^1^] 3315, 2914, 2852, 1444, 1382, 1137, 1049, 975, 900, 869, 786, 655. R_f_ 0.45 (60% EtOAc/hexanes, 1% Et_3_N) [CAM]. Mp 208–210 °C [lit. 206–208 °C][47]. The spectroscopic data were in agreement with previously reported data [15,48].

## 4. Conclusions

In summary, we developed and optimized a 15-step synthesis (11 steps LLS) of the potent alkaloid tomatidine in 15.2% overall yield starting from dinorcholanic lactone **5**. This methodology enabled us to produce 5.2 grams of tomatidine in a single pass using fine-tuned reaction sequences that require no flash chromatography purifications. We hope that this methodology can bridge the gap between what is synthetically feasible in the lab and what is industry-viable and that it can pave the way for easier access to this potent and promising biologically active natural product.

## Data Availability

Copies of NMR spectra for all synthesized compounds and abridged crystallographic data of **1** are available in the Supplementary Materials. Extended crystallographic data of **1** is available at the Cambridge Crystallographic Data Centre (ccdc.cam.ac.uk - deposition number 2090407).

## Supplementary Materials

The following are available online, Table S1: Crystal Data and Structure Refinement for **1,** Figures S1–S22: ^1^H and ^13^C NMR spectra of compounds **1**, **2**, OAc-**2**, **7**, **8**, **9a**, **11-13**, **15** and **16,** Figure S23: Vapor Diffusion Apparatus for the Crystallization of **1**.

## Dedication

This work is dedicated to the loving memory of the late Pr. Éric Marsault, an outstanding researcher, professional investigator, mentor, and friend who passed away too early.

## Sample Availability

No samples are available from the authors.
