# Supplementary material for: Concise Large-Scale Synthesis of Tomatidine, A Potent Antibiotic Natural Product
Source: Molecules. 2021 Oct 3;26(19):6008. doi: 10.3390/molecules26196008 (PMC8512692; doi:10.3390/molecules26196008)
Supplement: Supplementary file 1 [file molecules-26-06008-s001.zip › molecules-1398339-supplementary.pdf]

Supporting Information

Concise Large-Scale Synthesis of Tomatidine, a Potent Antibiotic Natural Product

Chad Normandin and Pierre-Luc Boudreault*

Table of Contents of the Supporting information

Table S1. Crystal data and structure refinement for **1** 2

Figure S1. ^{1}H NMR (400 MHz, $CDCl_3$) of **15** 3

Figure S2. ^{13}C NMR (400 MHz, $CDCl_3$) of **15** 3

Figure S3. ^{1}H NMR (400 MHz, $CDCl_3$) of **8** 4

Figure S4. ^{13}C NMR (400 MHz, $CDCl_3$) of **8** 4

Figure S5. ^{1}H NMR (400 MHz, $CDCl_3$) of **9a** 5

Figure S6. ^{13}C NMR (400 MHz, $CDCl_3$) of **9a** 5

Figure S7. ^{1}H NMR (400 MHz, $CDCl_3$) of **11** 6

Figure S8. ^{13}C NMR (400 MHz, $CDCl_3$) of **11** 6

Figure S9. ^{1}H NMR (400 MHz, $CDCl_3$) of **12** 7

Figure S10. ^{13}C NMR (400 MHz, $CDCl_3$) of **12** 7

Figure S11. ^{1}H NMR (400 MHz, $CDCl_3$) of **13** 8

Figure S12. ^{13}C NMR (100 MHz, $CDCl_3$) of **13** 8

Figure S13. ^{1}H NMR (400 MHz, $CDCl_3$) of **7** 9

Figure S14. ^{13}C NMR (100 MHz, $CDCl_3$) of **7** 9

Figure S15. ^{1}H NMR (400 MHz, $CDCl_3$) of **2** 10

Figure S16. ^{13}C NMR (100 MHz, $CDCl_3$) of **2** 10

Figure S17. ^{1}H NMR (400 MHz, $CDCl_3$) of OAc-**2** 11

Figure S18. ^{13}C NMR (100 MHz, $CDCl_3$) of OAc-**2** 11

Figure S19. ^{1}H NMR (400 MHz, $CDCl_3$) of **16** 12

Figure S20. ^{13}C NMR (100 MHz, $CDCl_3$) of **16** 12

Figure S21. ^{1}H NMR (400 MHz, $CDCl_3$) of **1** 13

Figure S22. ^{13}C NMR (100 MHz, $CDCl_3$) of **1** 13

Figure S23. Vapor Diffusion Apparatus for the Crystallization of **1** 14

Table S1. Crystal Data and Structure Refinement for **1**

Empirical formula	$C_{27}H_{45}NO_2 \bullet CH_3OH$
Formula weight	447.68 g / mol
Temperature	173 K
Wavelength	1.54178 Å
Crystal system	Monoclinic
a	13.2306(5) Å
b	7.4199(2) Å
c	14.8312(5) Å
α	90 °
β	115.7860(10) °
γ	90 °
Volume	1311.00(8) $Å^3$
Z, Calculated density	1.134 g/cm^3
Absorption coefficient	0.554 mm^{-1}
F(000)	496
Crystal size	0.430 x 0.592 x 0.750 mm
Theta Range for data collection	3.31 to 71.87°
Index ranges	-15<=h<=16, -9<=k<=8, -17<=l<=18
Reflections collected/ unique	13514
Completeness	97.1 %
Absorption correction	Multi-Scan
Max. and min. transmission	0.7530 and 0.4570
Refinement method	Full-matrix least-squares on F^2
Data/ restraints/ parameter	4556 / 2 / 301
Goodness-of-fit on F2	1.056
Final R (I > 2σ (I))	R1 = 0.0544, wR2 = 0.1483
R indices (all data)	R1 = 0.0579, wR2 = 0.1534
Absolute structure parameter	0.02(16)
Largest diff. peak and hole (e. Å-3)	0.265 and -0.238 $eÅ^{-3}$

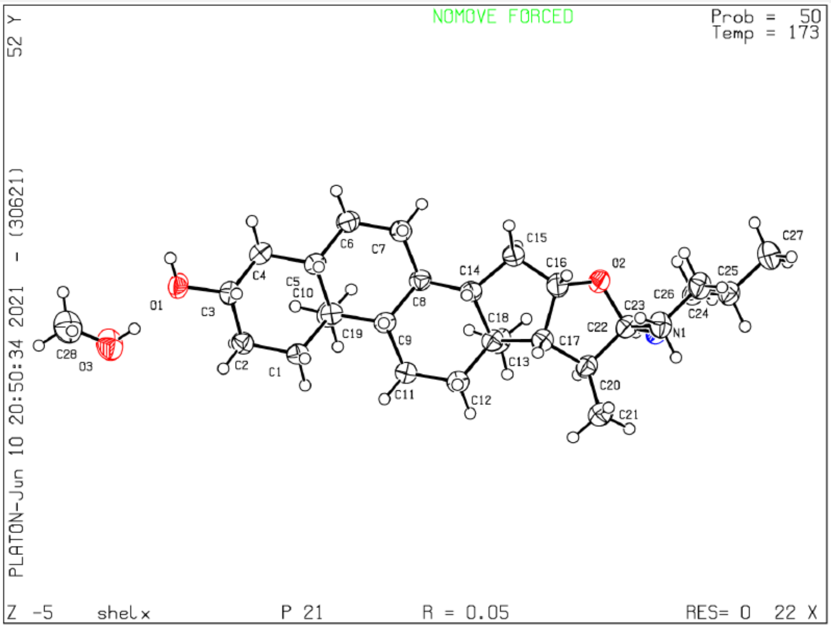

The structure was deposited at the Cambridge Crystallographic Data Centre with the deposition number #2090407.

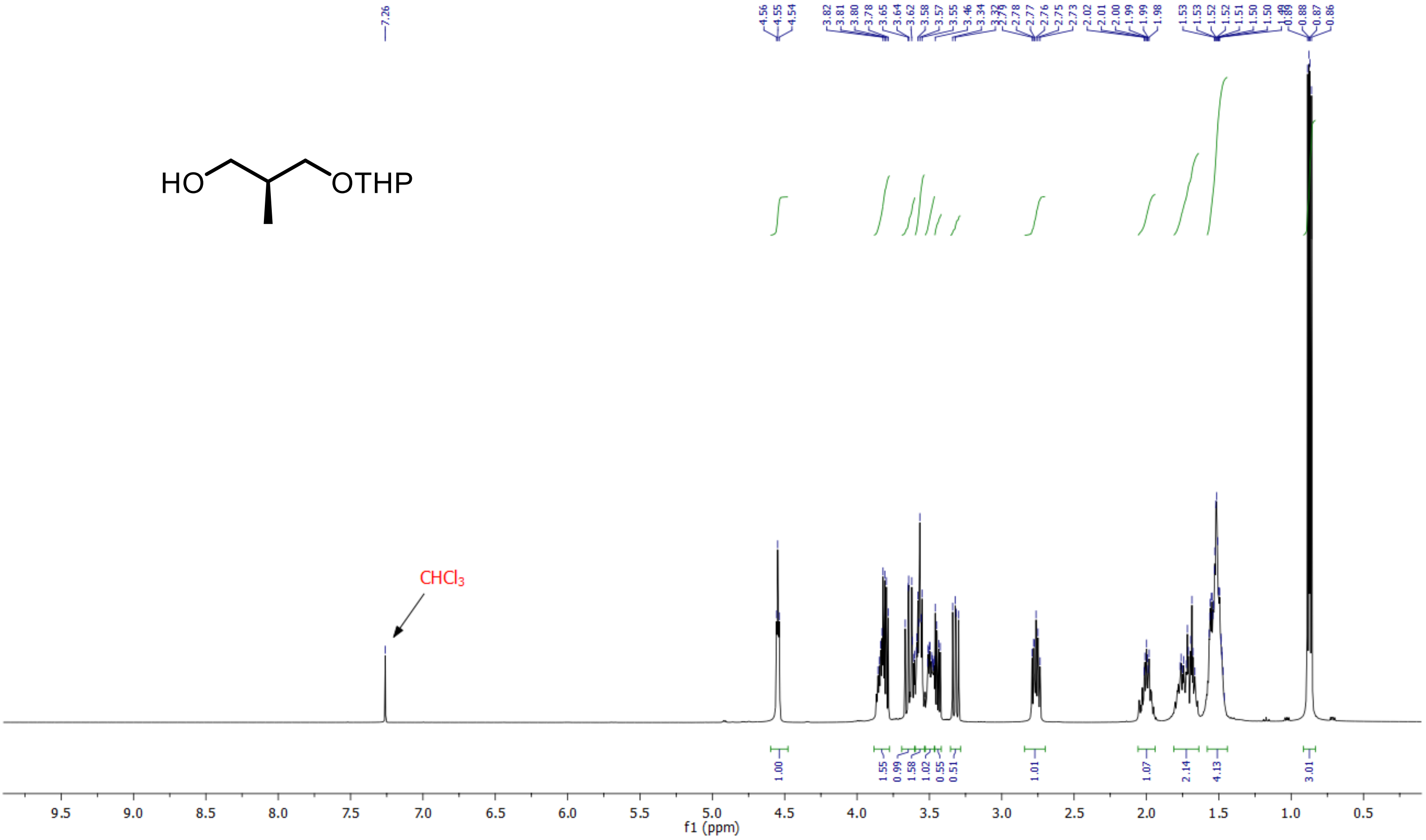

Figure S1. ^{1}H NMR (400 MHz, $CDCl_3$) of **15**

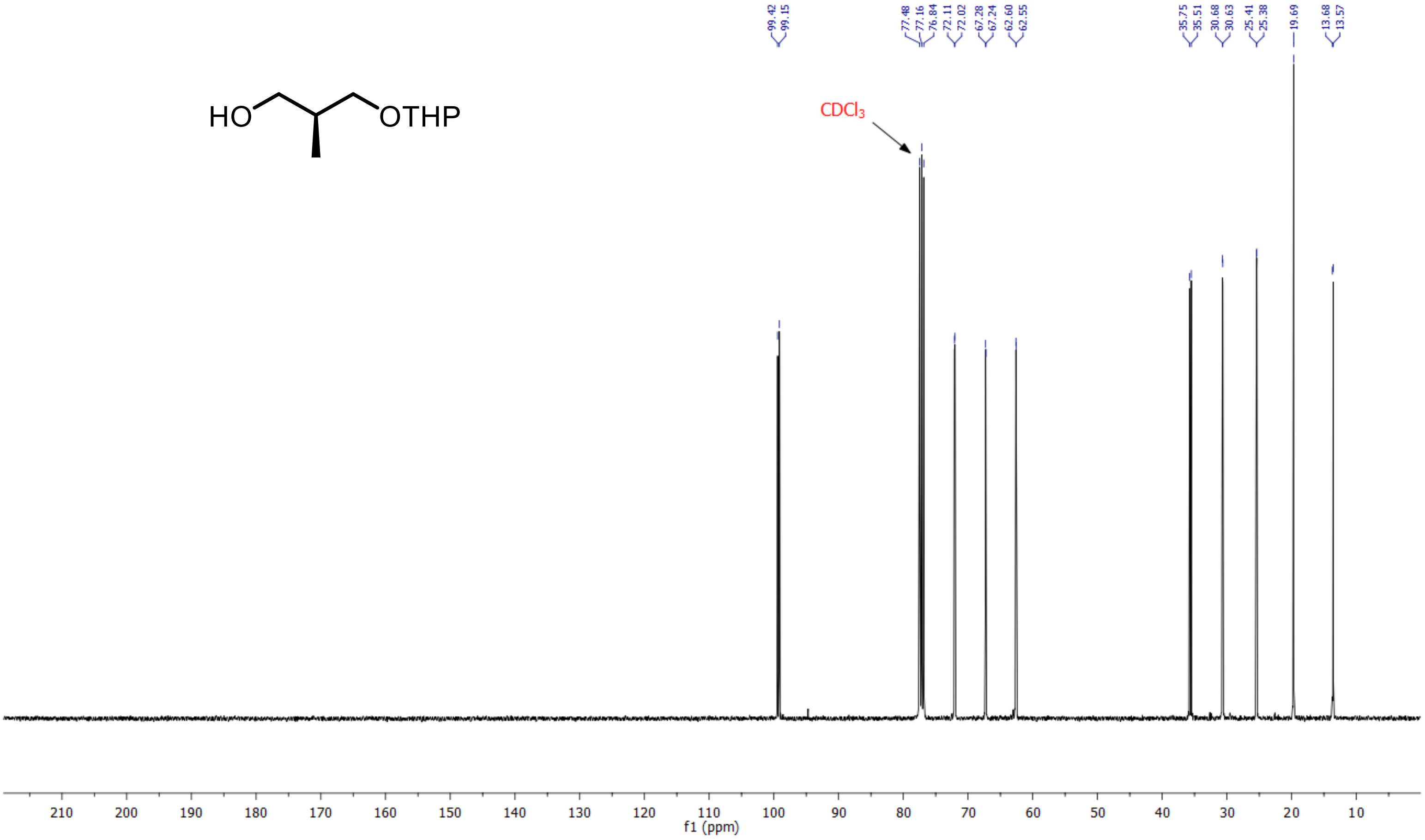

Figure S2. ^{13}C NMR (400 MHz, $CDCl_3$) of **15**

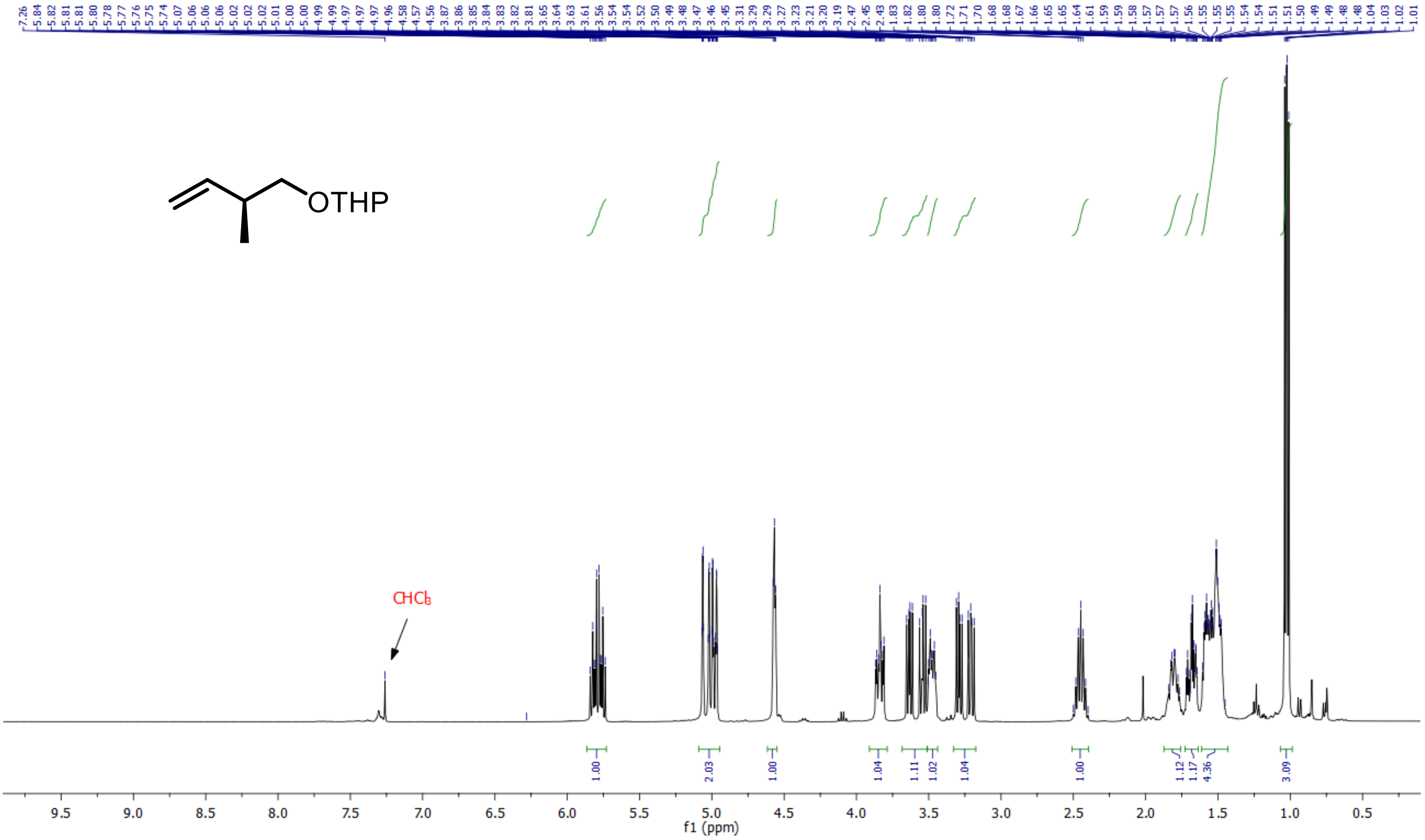

Figure S3. ^{1}H NMR (400 MHz, $CDCl_3$) of **8**

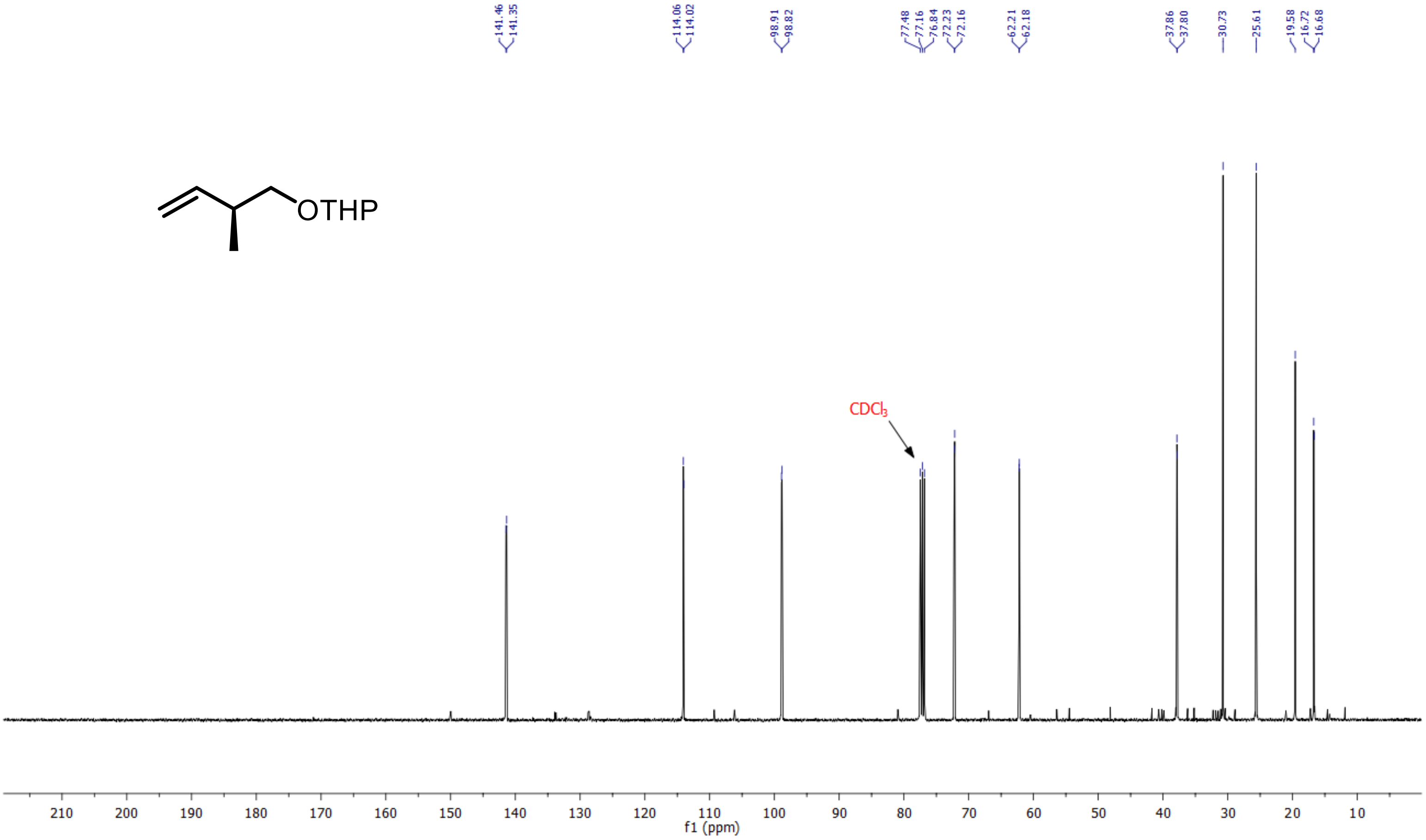

Figure S4. ^{13}C NMR (400 MHz, $CDCl_3$) of **8**

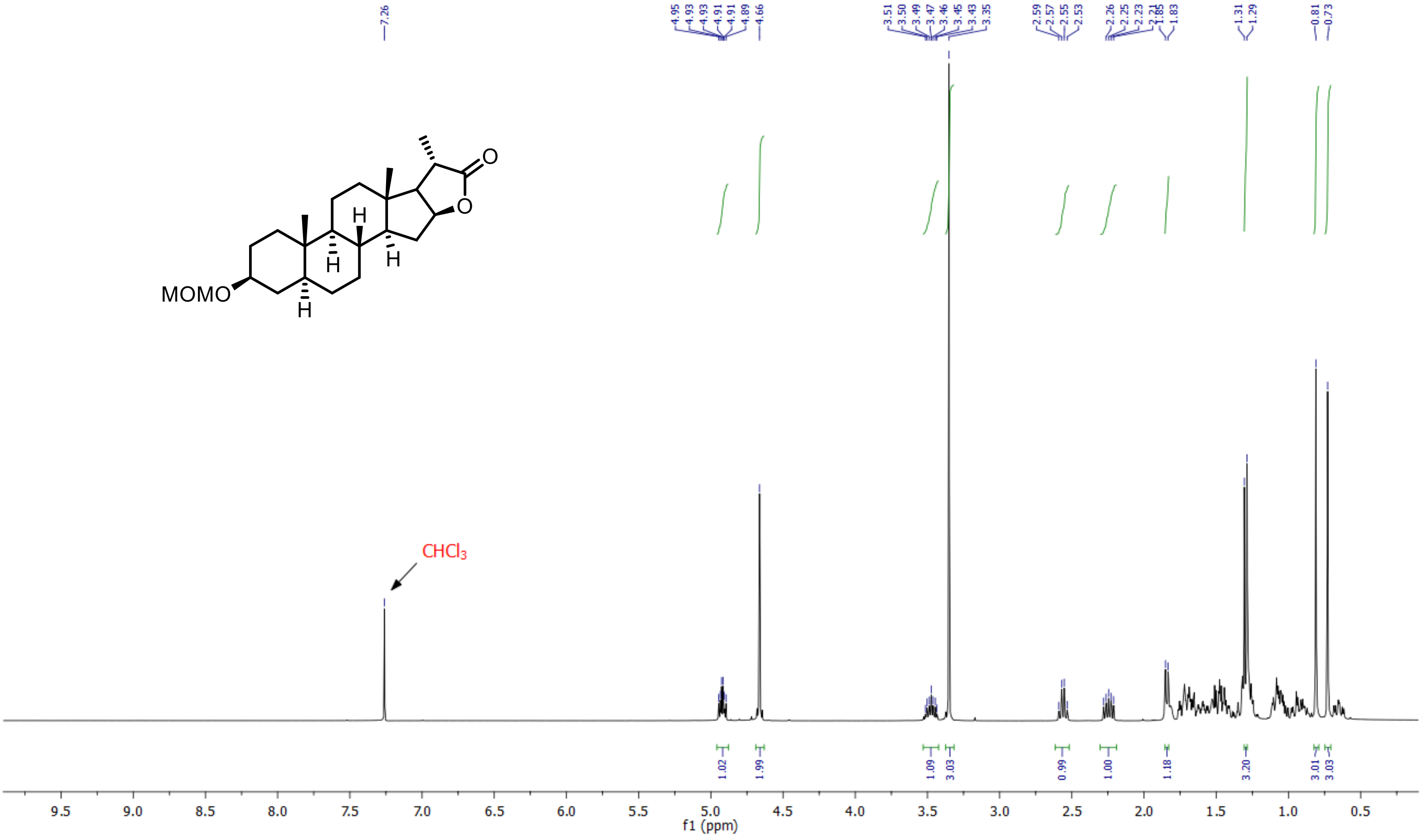

Figure S5. ^{1}H NMR (400 MHz, $CDCl_3$) of **9a**

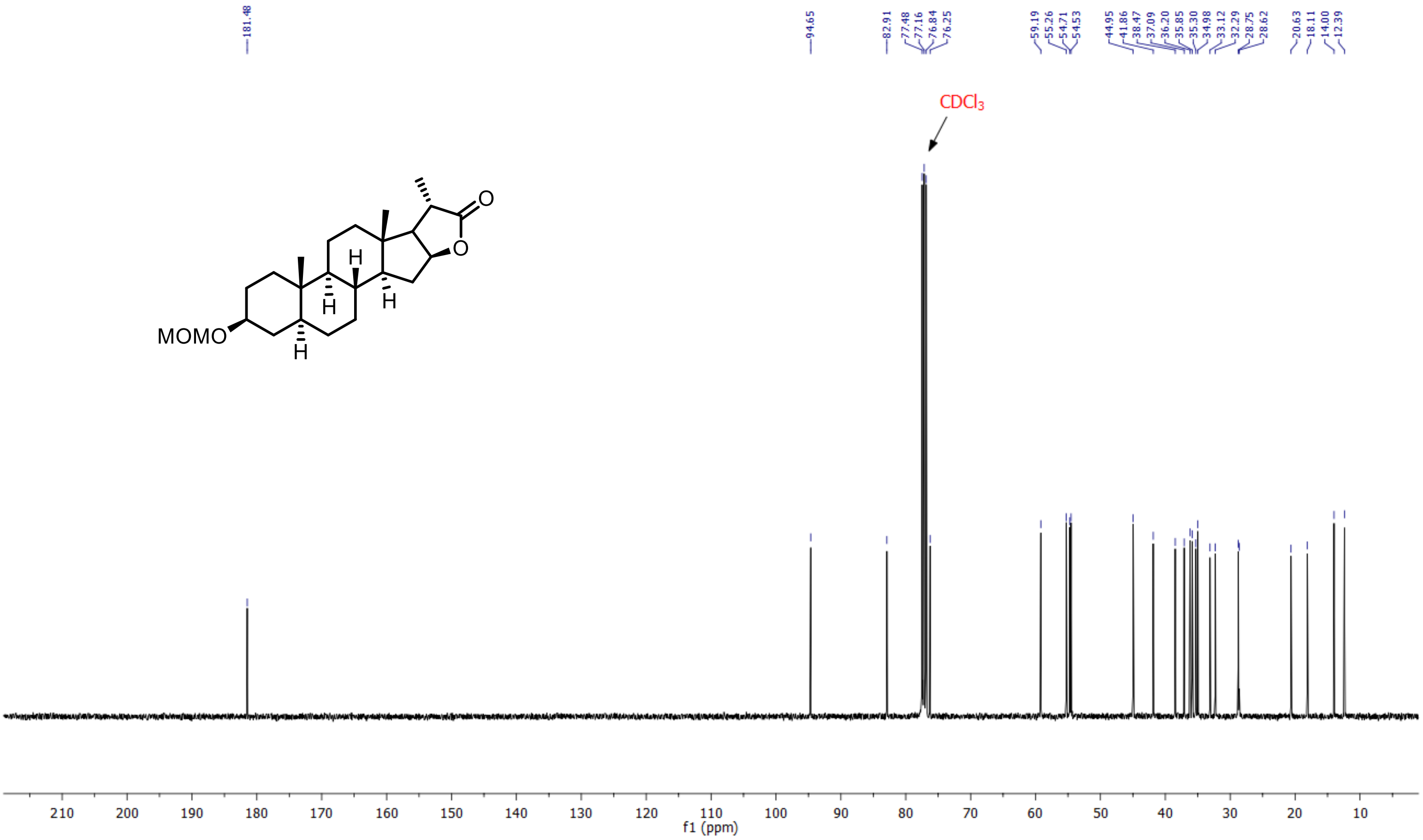

Figure S6. ^{13}C NMR (400 MHz, $CDCl_3$) of **9a**

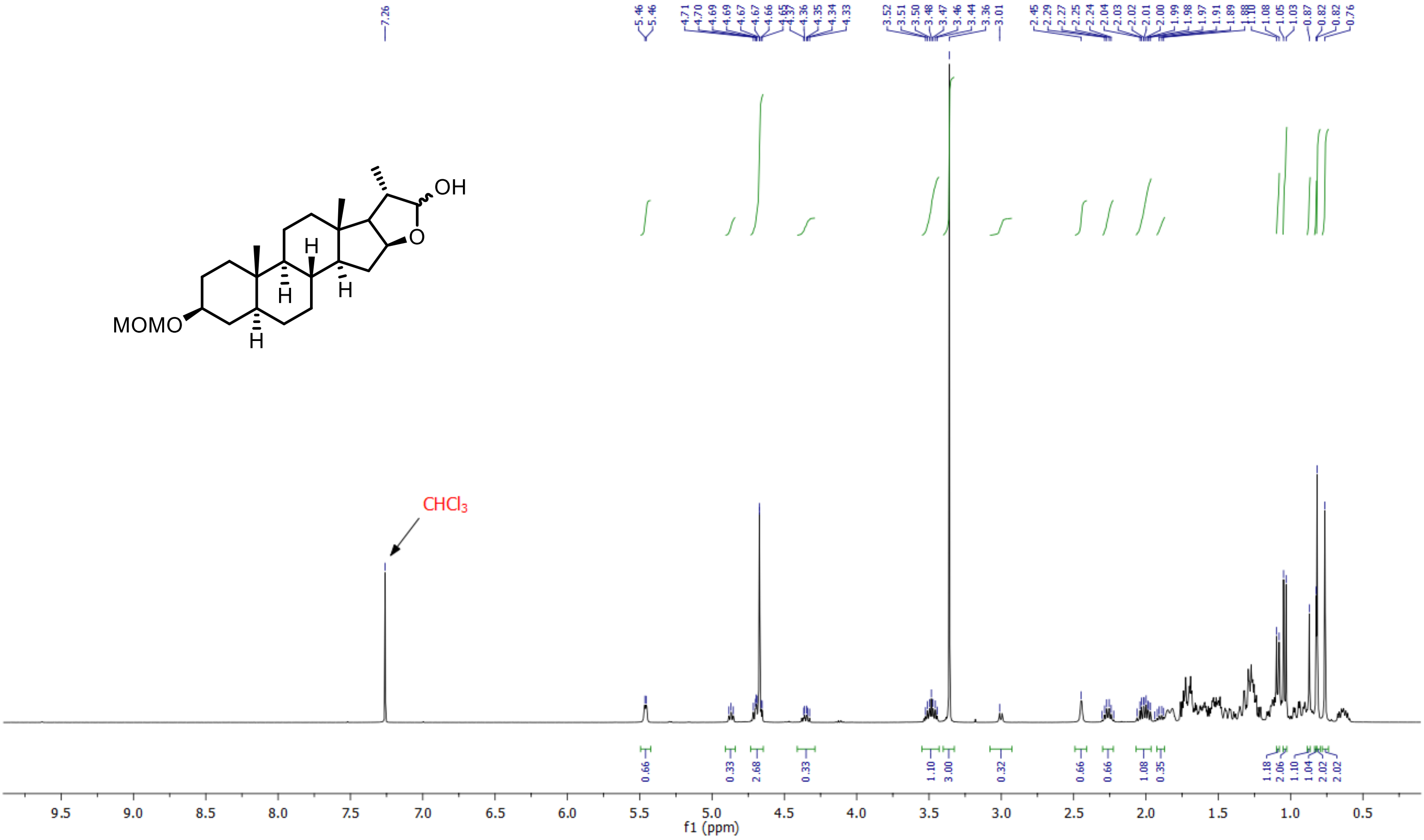

Figure S7. ^{1}H NMR (400 MHz, $CDCl_3$) of **11**

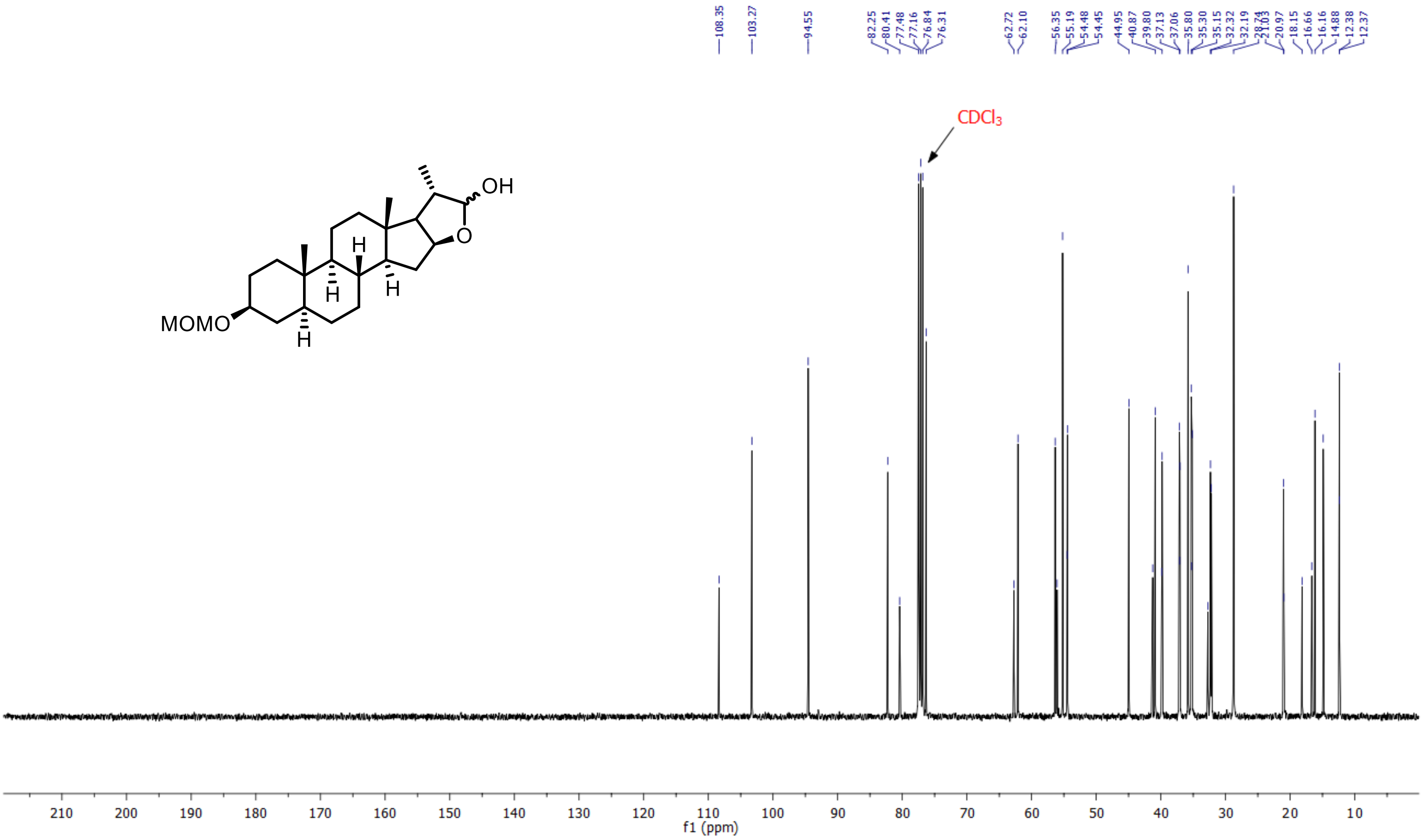

Figure S8. ^{13}C NMR (400 MHz, $CDCl_3$) of **11**

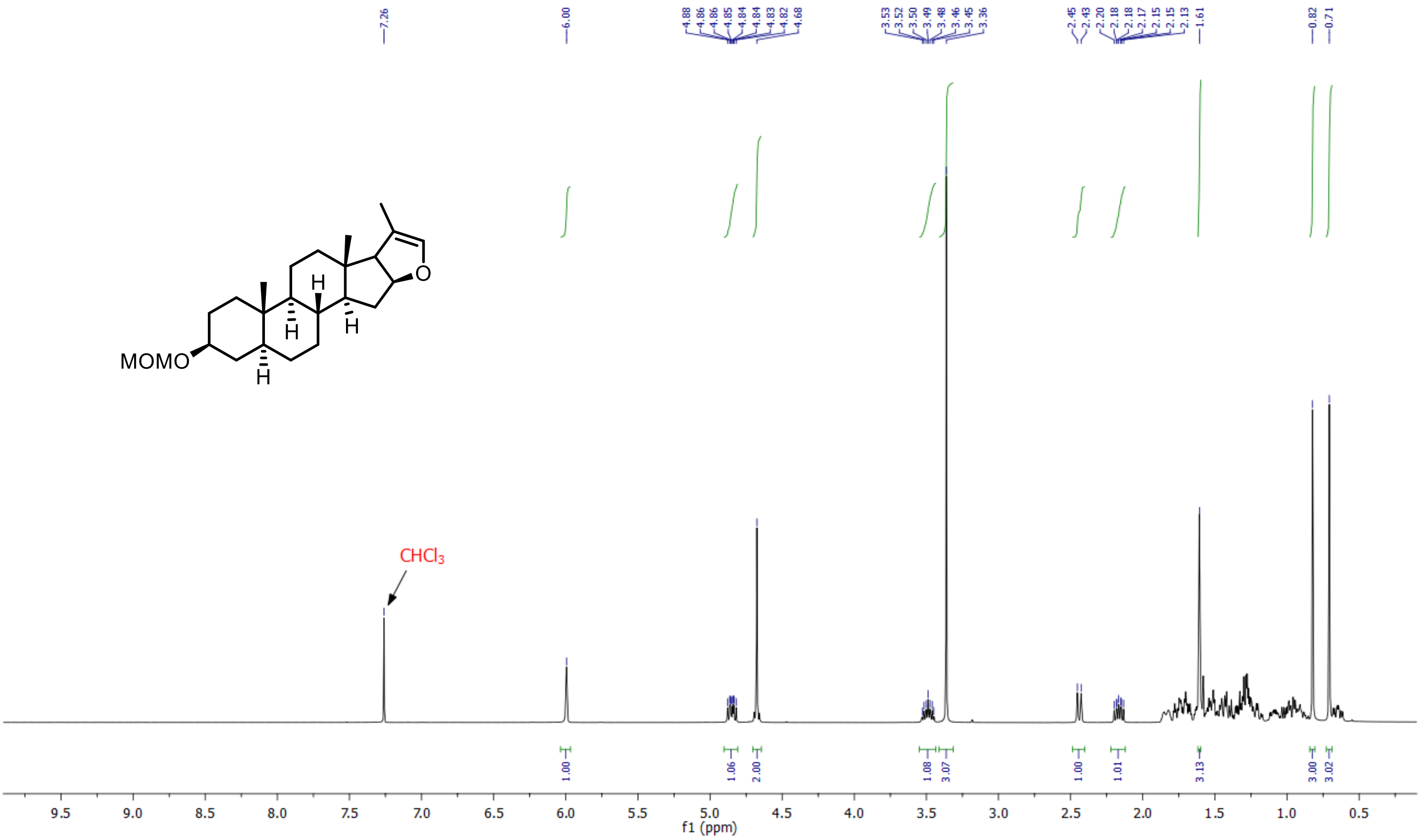

Figure S9. ^{1}H NMR (400 MHz, $CDCl_3$) of **12**

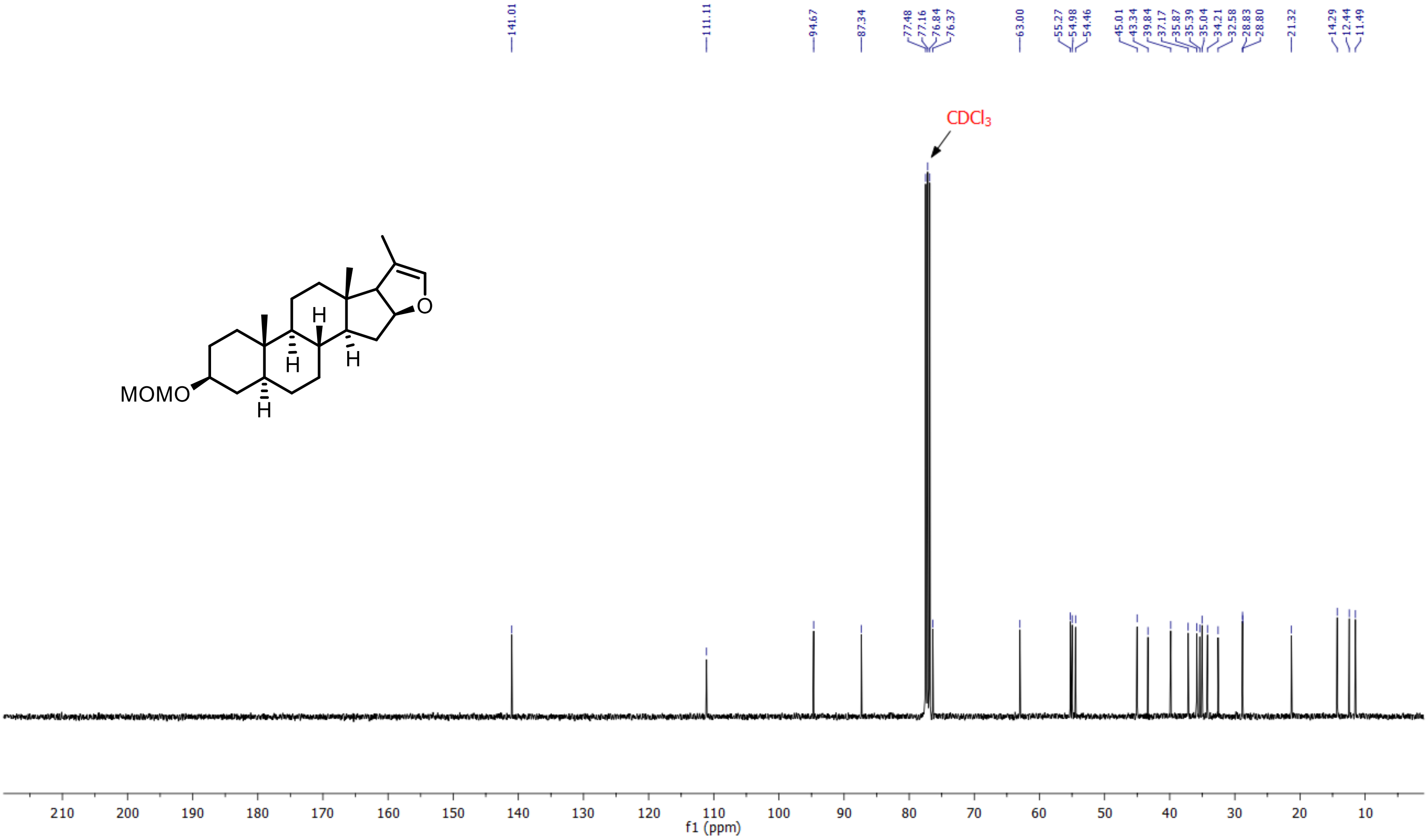

Figure S10. ^{13}C NMR (400 MHz, $CDCl_3$) of **12**

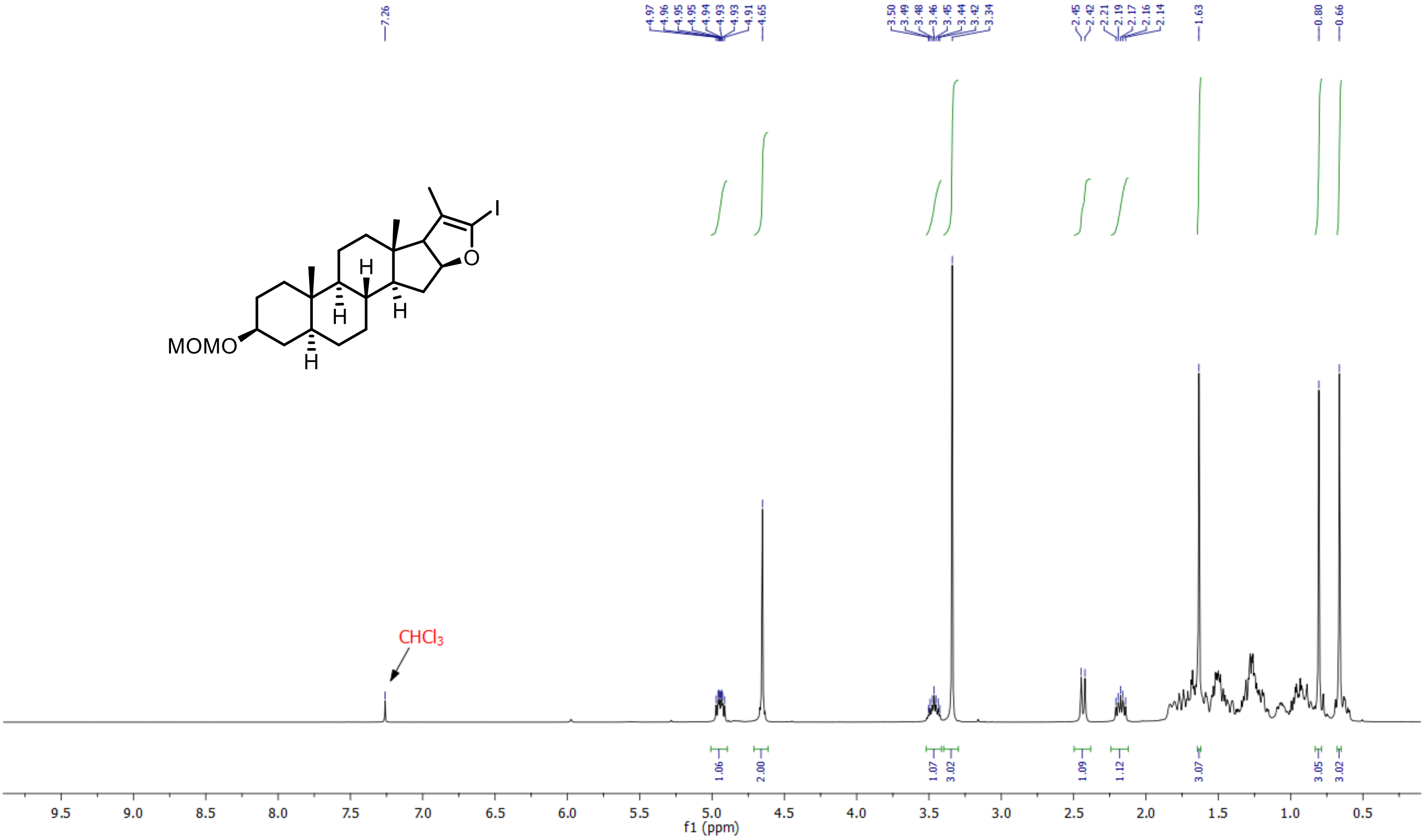

Figure S11. ^{1}H NMR (400 MHz, $CDCl_3$) of **13**

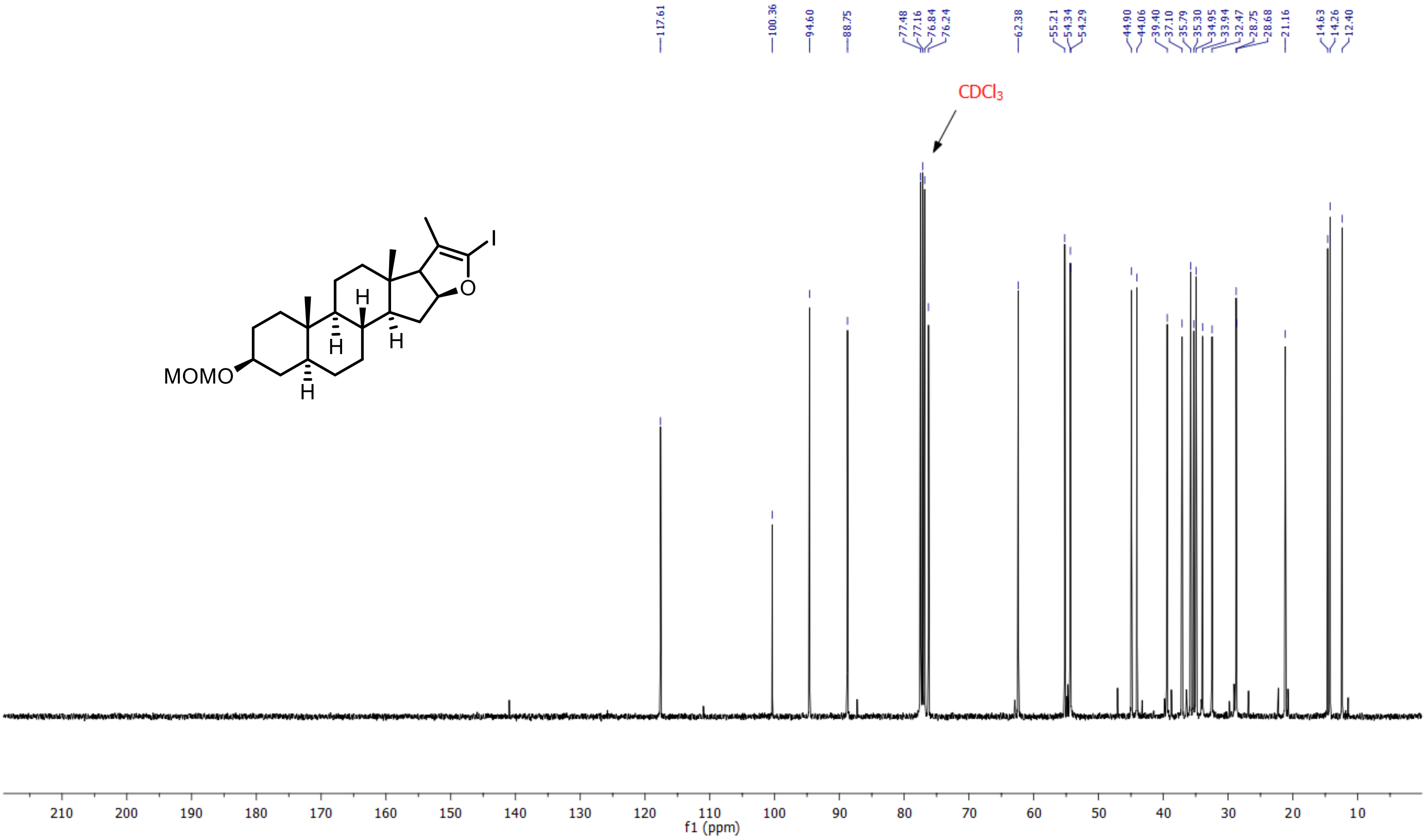

Figure S12. ^{13}C NMR (100 MHz, $CDCl_3$) of **13**

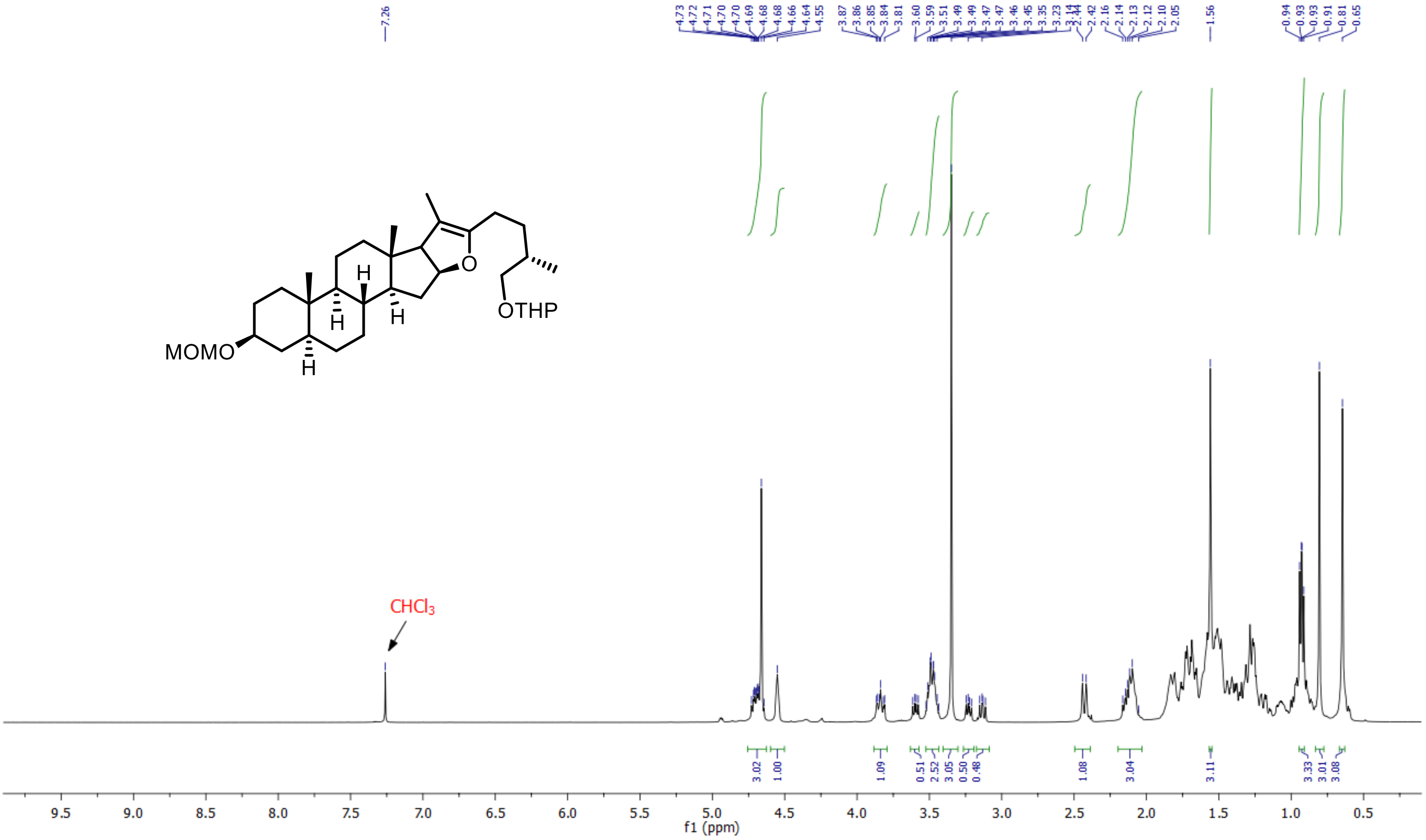

Figure S13. ^{1}H NMR (400 MHz, $CDCl_3$) of **7**

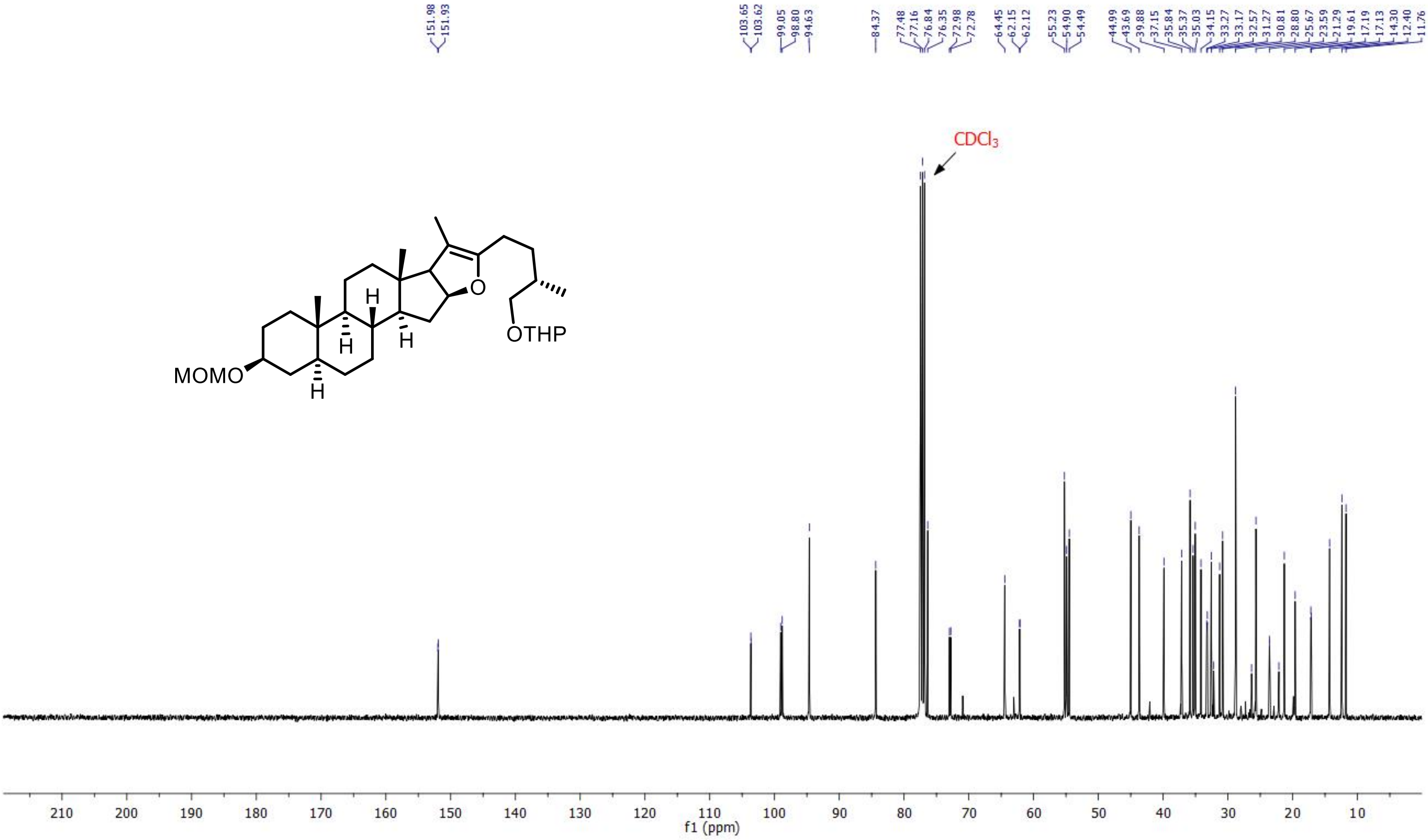

Figure S14. ^{13}C NMR (100 MHz, $CDCl_3$) of **7**

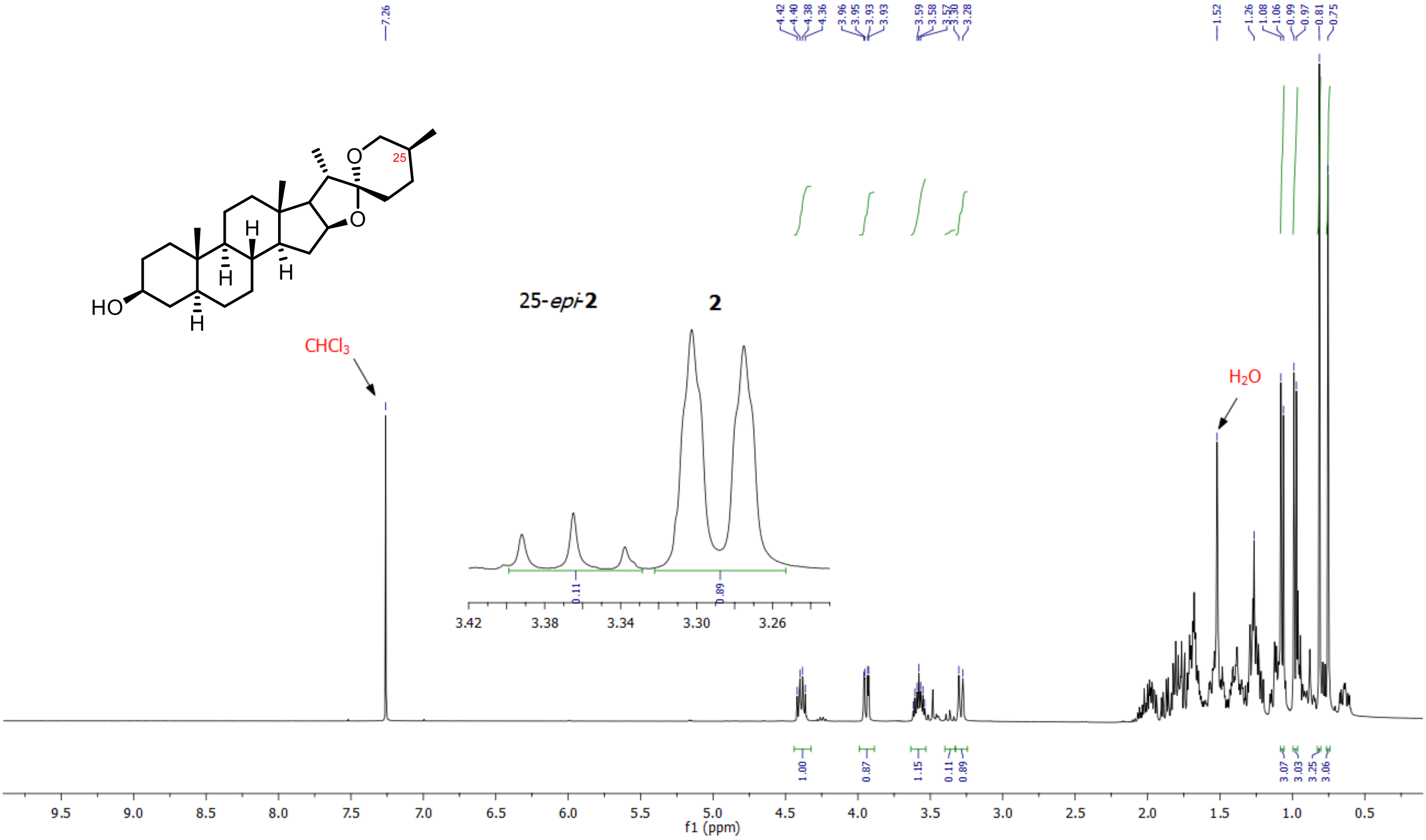

Figure S15. ^{1}H NMR (400 MHz, $CDCl_3$) of **2**

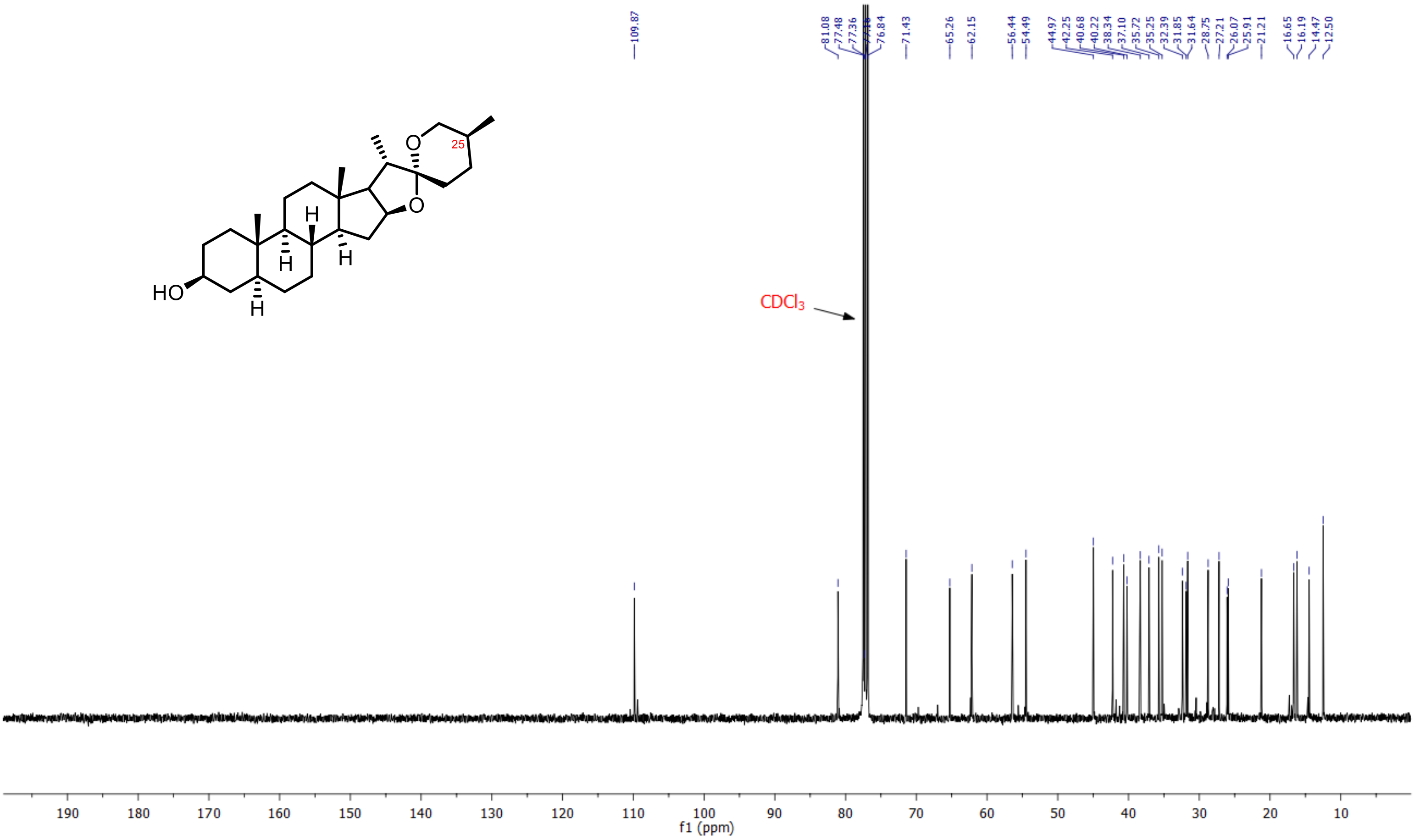

Figure S16. ^{13}C NMR (100 MHz, $CDCl_3$) of **2**

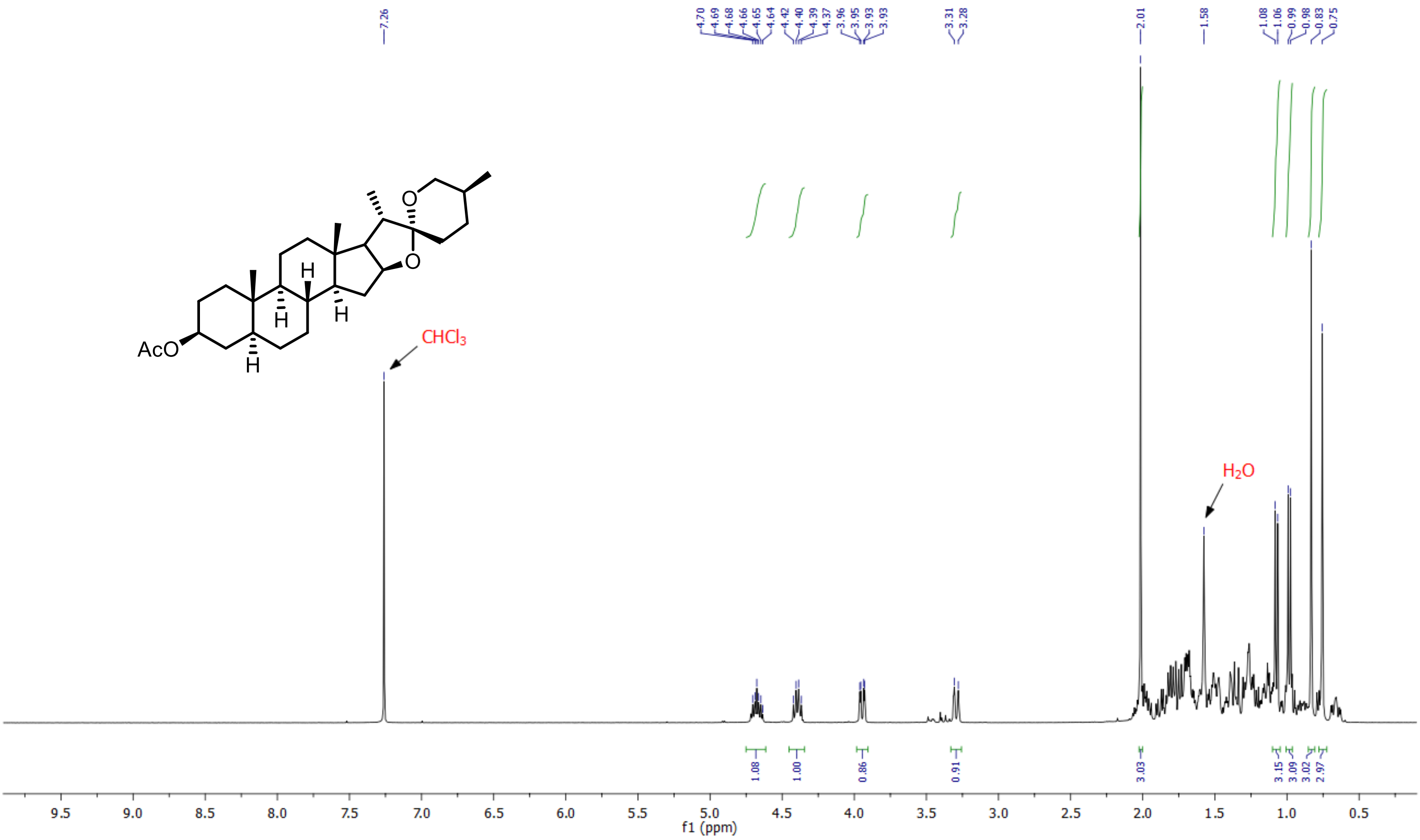

Figure S17. ^{1}H NMR (400 MHz, $CDCl_3$) of OAc-**2**

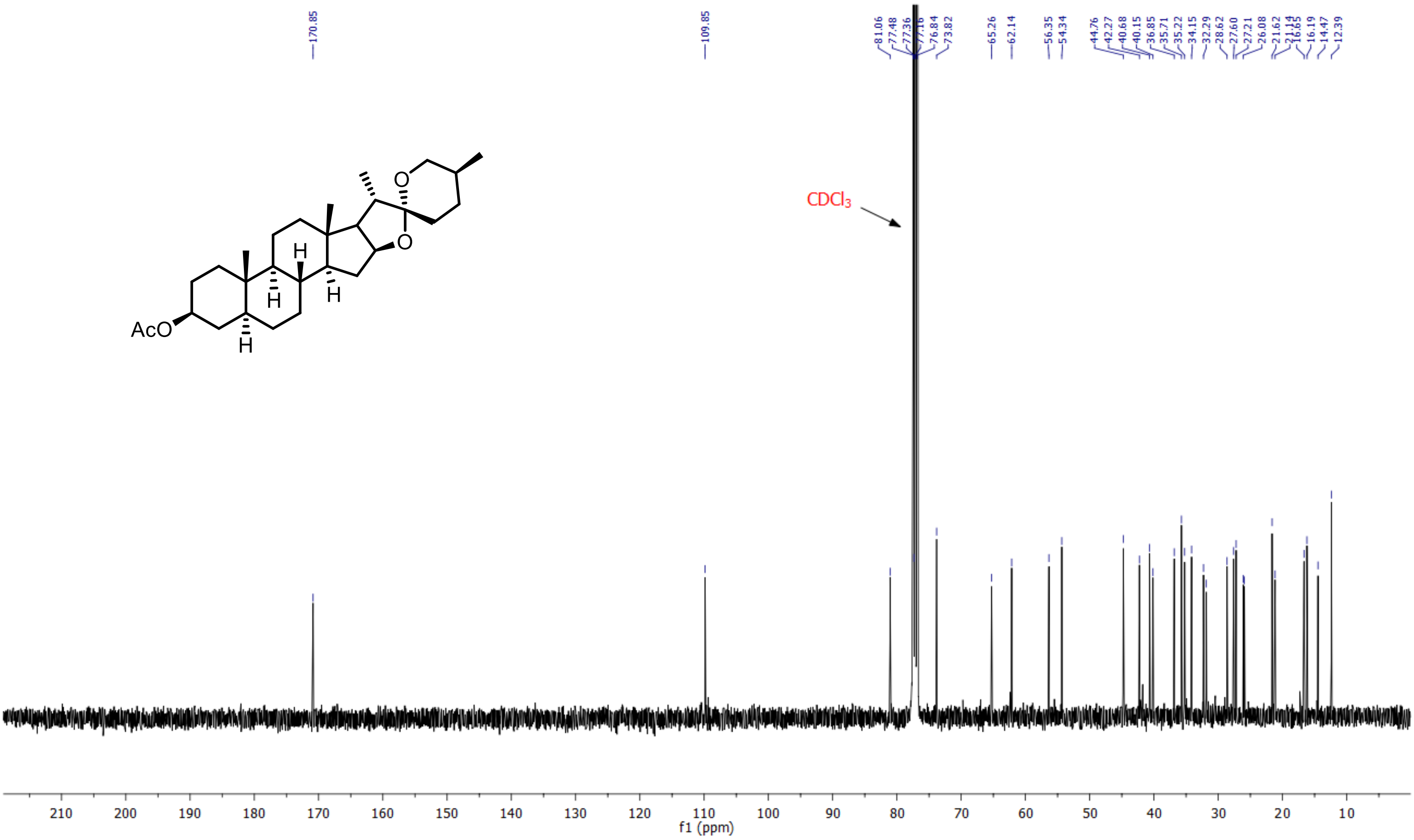

Figure S18. ^{13}C NMR (100 MHz, $CDCl_3$) of OAc-**2**

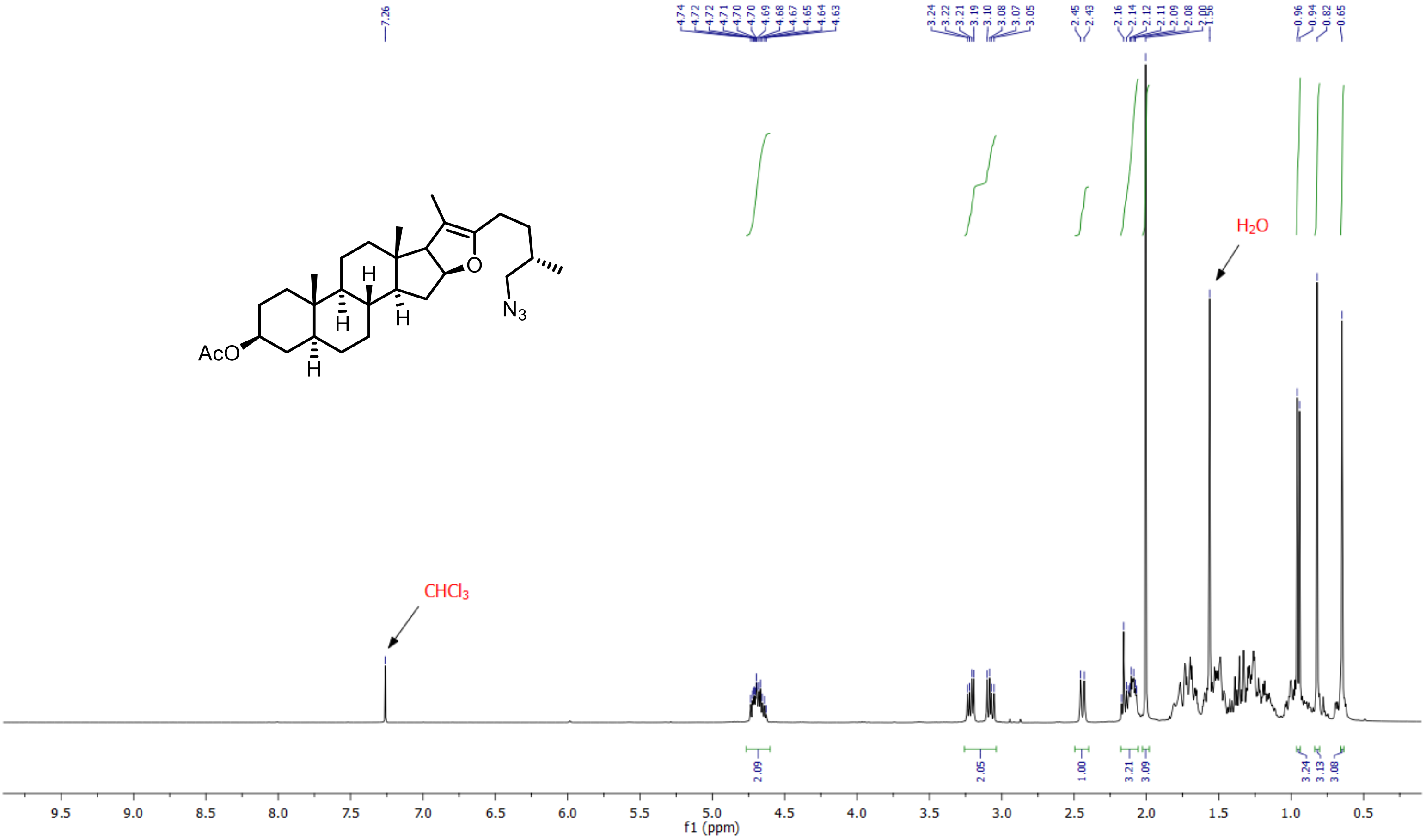

Figure S19. ^{1}H NMR (400 MHz, $CDCl_3$) of **16**

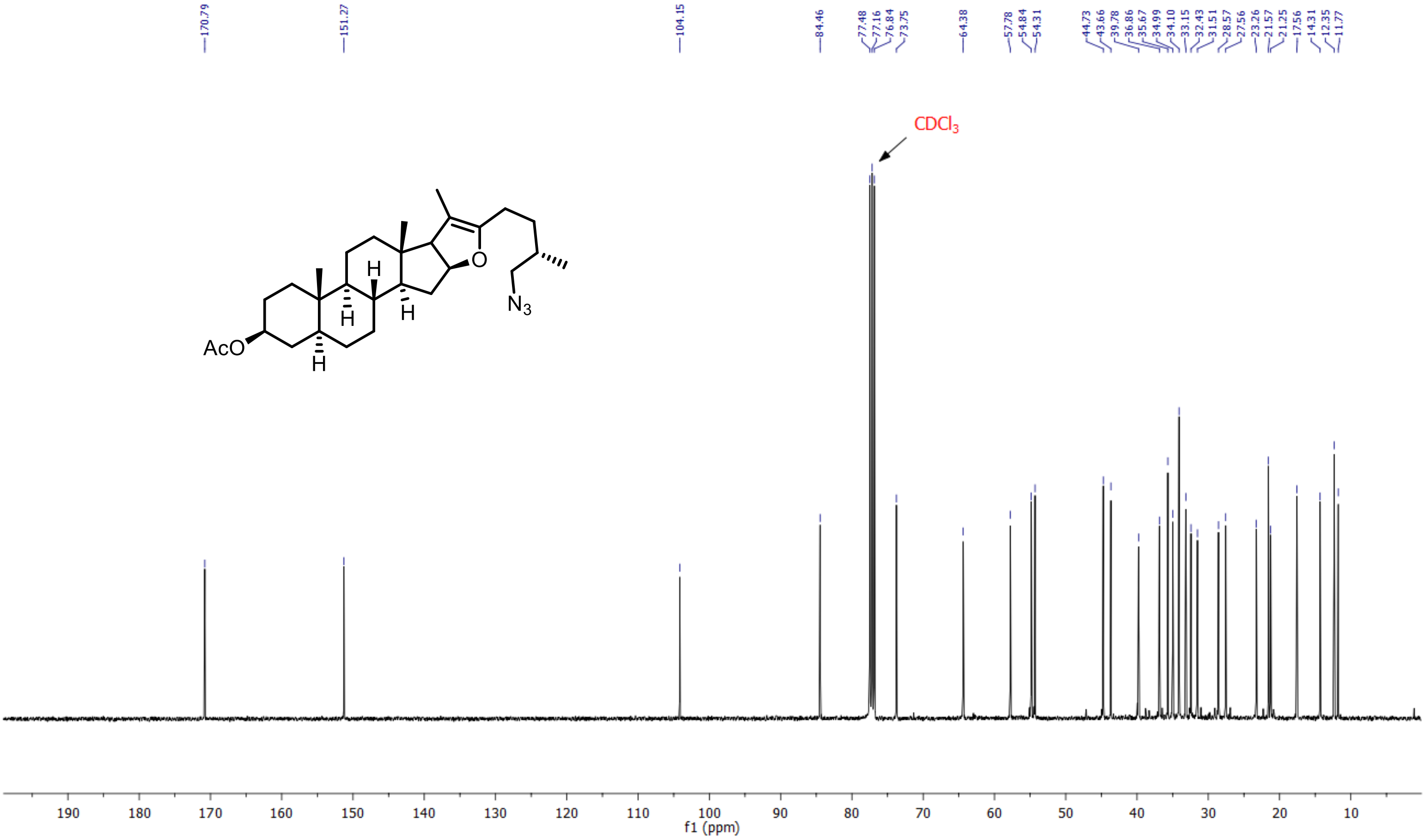

Figure S20. ^{13}C NMR (100 MHz, $CDCl_3$) of **16**

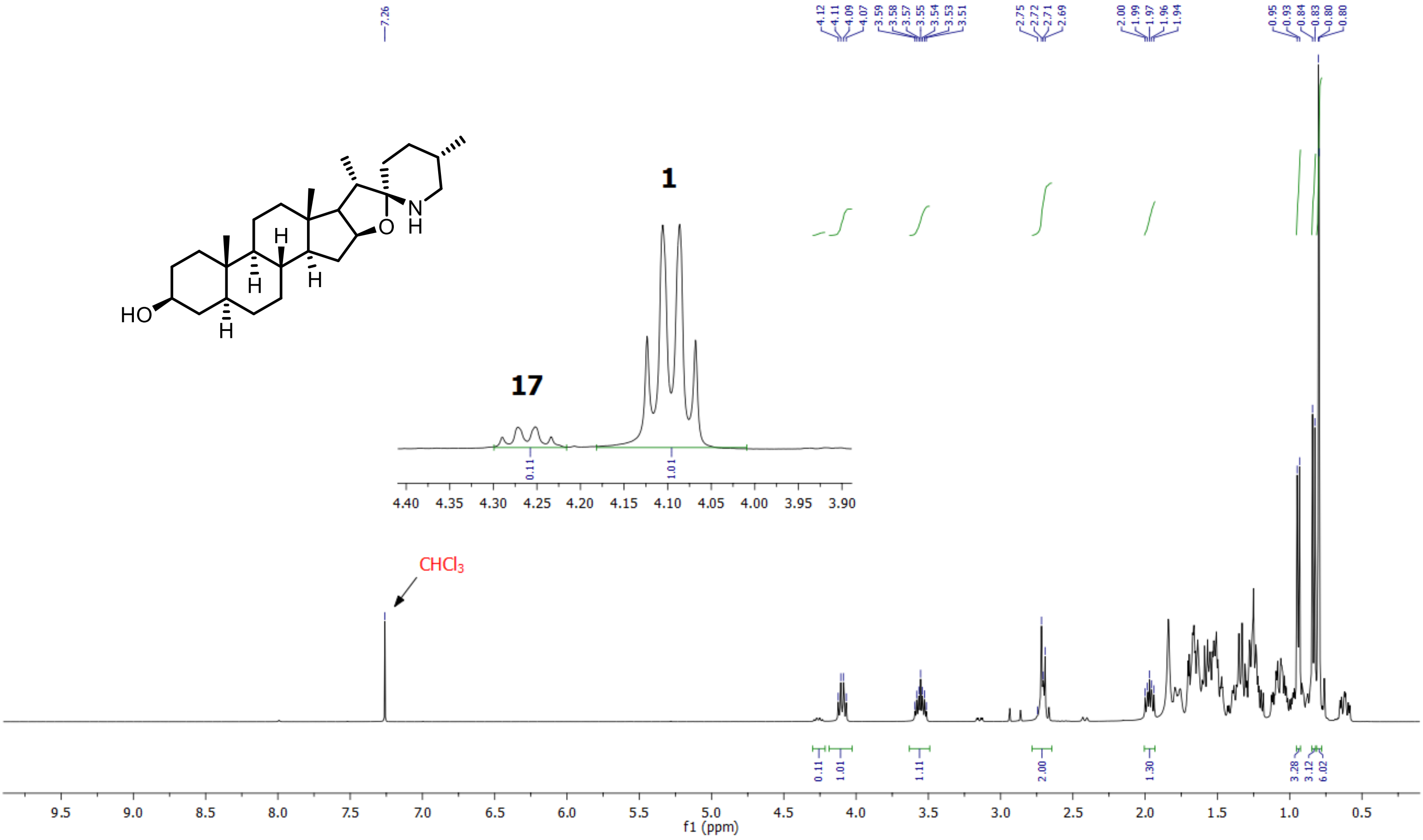

Figure S21. ^{1}H NMR (400 MHz, $CDCl_3$) of **1**

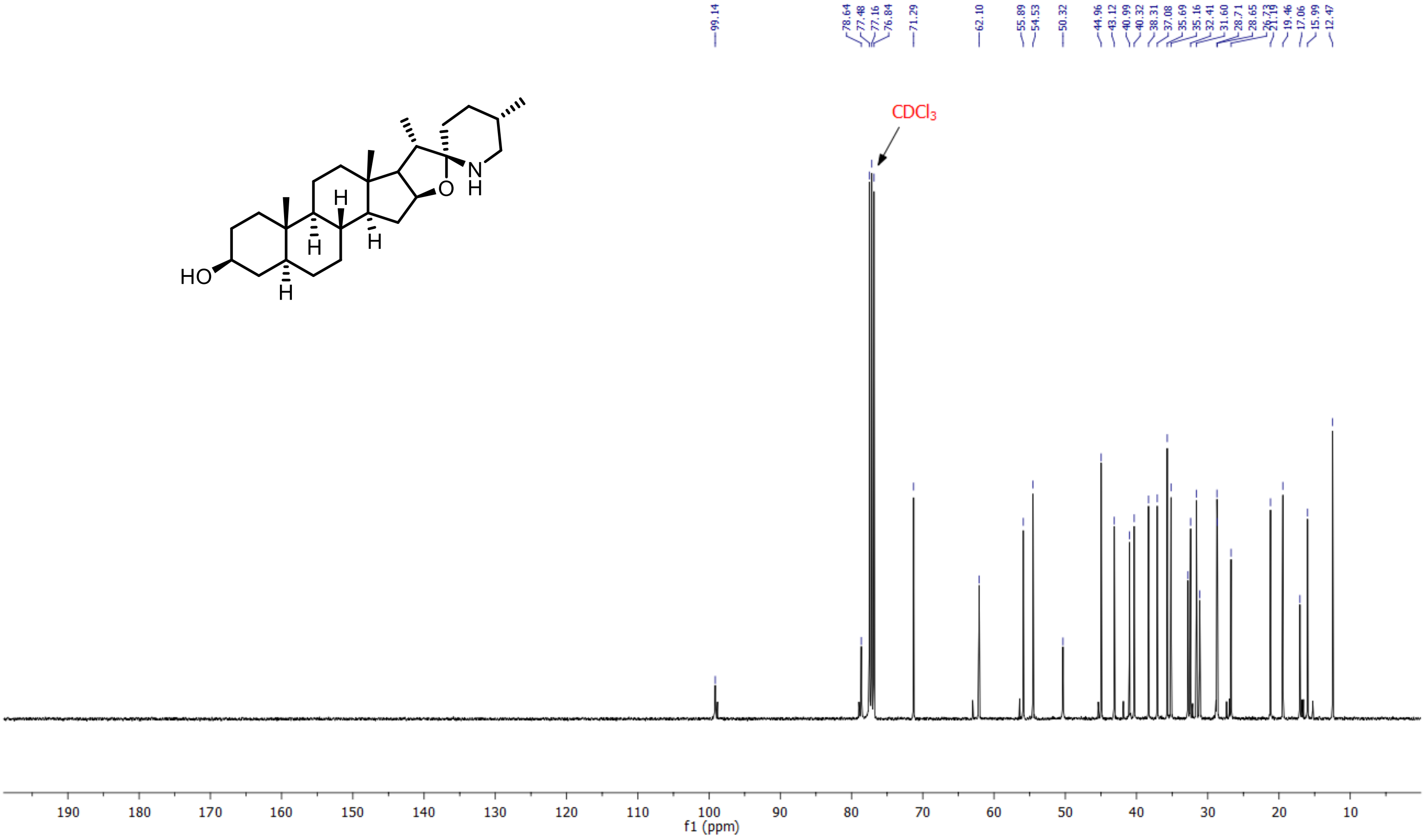

Figure S22. ^{13}C NMR (100 MHz, $CDCl_3$) of **1**

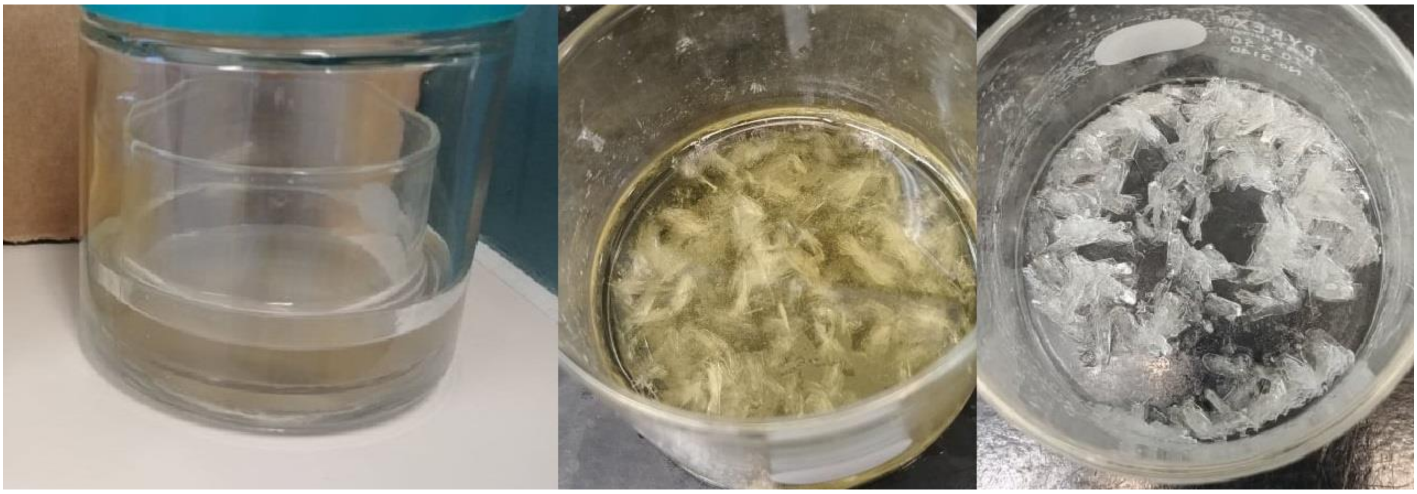

Figure S23. Vapor Diffusion Apparatus for the Crystallization of **1**
